# Does grasping capacity influence object size estimates? It depends on the context

**DOI:** 10.3758/s13414-017-1344-3

**Published:** 2017-06-21

**Authors:** Elizabeth S. Collier, Rebecca Lawson

**Affiliations:** Department of Experimental Psychology, University of Liverpool, Eleanor Rathbone Building Bedford Street South, Liverpool, L69 7ZA UK

**Keywords:** Perception, Action, Demand characteristics, Vision, Task demands

## Abstract

Linkenauger, Witt, and Proffitt (*Journal of Experimental Psychology: Human Perception and Performance, 37*(5), 1432–1441, 2011, Experiment 2) reported that right-handers estimated objects as smaller if they intended to grasp them in their right rather than their left hand. Based on the action-specific account, they argued that this scaling effect occurred because participants believed their right hand could grasp larger objects. However, Collier and Lawson (*Journal of Experimental Psychology: Human Perception and Performance, 43*(4), 749–769, 2017) failed to replicate this effect. Here, we investigated whether this discrepancy in results arose from demand characteristics. We investigated two forms of demand characteristics: altering responses following conscious *hypothesis guessing* (Experiments 1 and 2), and subtle influences of the experimental *context* (Experiment 3). We found no scaling effects when participants were given instructions which implied the expected outcome of the experiment (Experiment 1), but they were obtained when we used unrealistically explicit instructions which gave the exact prediction made by the action-specific account (Experiment 2). Scaling effects were also found using a context in which grasping capacity could seem relevant for size estimation (by asking participants about the perceived graspability of an object immediately before asking about its size on every trial, as was done in Linkenauger et al., 2011; Experiment 2). These results suggest that demand characteristics due to context effects could explain the scaling effects reported in Experiment 2 of Linkenauger et al. (2011), rather than either hypothesis guessing, or, as proposed by the action-specific account, a change in the perceived size of objects.

**Electronic supplementary material:**

The online version of this article (doi:10.3758/s13414-017-1344-3) contains supplementary material, which is available to authorized users.

The term *action capacity* refers to our ability to successfully perform actions. It is restricted by the morphology and capabilities of our bodies (Adolph & Berger, 2006; Proffitt & Linkenauger, 2013). Given the tight coupling between perception and action (Clark, 1999; Gibson, 1979; Warren, 1984), it has been suggested that action capacity can directly influence visual perception (Proffitt & Linkenauger, 2013; Witt, 2011, 2016). Specifically, the action-specific account of perception suggests that our perception of the spatial properties of the environment scales according to our action capacity (Proffitt, 2006; Proffitt & Linkenauger, 2013). For example, reaching with a tool that increases maximum reach can influence the estimated distance to a target (Witt, Proffitt & Epstein, 2005). Witt et al. (2005) found that targets which were out of reach without the tool were estimated as closer after reaching to them with the tool.

Action-specific scaling effects suggest that perception may be cognitively penetrable—so perception can be directly influenced by higher-level cognition. If action-specific scaling effects truly reflect changes in what is perceived in this strong sense, then this has major implications for standard, modular theories of vision, which hold that perception is encapsulated and separate from cognition (Pylyshyn, 1999; for a recent review, see Firestone & Scholl, 2015). However, a major debate concerning the action-specific account is whether the observed scaling effects reflect judgement rather than perception (Collier & Lawson, 2017; Durgin et al., 2009; Durgin, Klein, Spiegel, Strawset & Williams, 2012; Firestone & Scholl, 2014; Zelaznik & Forney, 2016; for reviews, see Firestone, 2013; Firestone & Scholl, 2015; Philbeck & Witt, 2015; Proffitt & Linkenauger, 2013; Witt, 2011, 2016). Specifically, participants’ responses may not reflect differences in what they actually perceive; rather, their spatial estimates may be affected by nonperceptual influences such as their beliefs about the purpose of the experiment.

This possibility has been demonstrated experimentally. In a famous study supporting the action-specific account, hills were reported as steeper when observers wore a heavy backpack (Bhalla & Proffitt, 1999). However, Durgin et al. (2009) found that if participants were told that the backpack they wore contained equipment for monitoring their ankle muscles, their estimates of hill slant did not differ from participants who did not wear the backpack. This finding suggests that participants who were not given a reason for wearing the backpack may have deduced that the backpack was supposed to influence their estimates of hill slant and adjusted their responses accordingly. Similarly, Firestone and Scholl (2014) tested whether the finding that apertures were estimated as narrower when participants held a horizontal rod that was wider than their body (Stefanucci & Geuss, 2009) reflected a true perceptual change or demand characteristics. Firestone and Scholl (2014) found that when participants were given a convincing reason for holding the rod, their estimates of aperture width did not differ from participants who did not hold the rod. These results suggest that if participants are not given an explanation for a salient manipulation, they may attempt to figure out the experimental hypothesis and that this, in turn, can influence their responses.

Together, the results of Durgin et al. (2009), Firestone and Scholl (2014; see also Woods, Philbeck & Danoff, 2009) suggest that demand characteristics could explain a number of action-specific scaling effects. Demand characteristics broadly refer to factors in an experimental setting which affect participants’ responses (Orne, 1962). We will term the form of demand characteristics investigated by these authors *hypothesis guessing,* where participants try to work out the expected results of the experiment and consciously adjust their responses accordingly.

Such demand characteristics cannot, though, easily explain all action-specific effects (for some recent reviews, see Philbeck & Witt, 2015; Witt, 2016). For example, Taylor-Covill and Eves (2016) found that overweight individuals estimated staircases as steeper than did healthy-weight individuals. These results are difficult to explain in terms of hypothesis guessing (see also Witt & Sugovic, 2013). Although participants probably knew their own weight, they were unlikely to intuit that this was expected to influence what they perceived spatially, particularly given that Taylor-Covill and Eves (2016) recorded the participant's weight only after they had made their estimates of slant.

Another form of demand characteristics could, though, influence performance without participants necessarily realising it, namely *context effects* due to the experimental setting or procedure. For example, performing two tasks in quick succession could create a context which implies that the two tasks are related in some meaningful way. In an example from the action-specific literature, Linkenauger, Witt, and Proffitt (2011, Experiment 2) reported that objects to-be-grasped in the right hand were estimated as smaller than objects to-be-grasped in the left hand. They claimed that this occurred because right-handers perceive their right hand as larger than their left hand, and so objects appear more graspable, and therefore smaller, when they intend to grasp them with their right hand. However, participants in Experiment 2 of Linkenauger et al. (2011) estimated both the graspability and size of objects on every trial. Asking participants about an object’s graspability immediately before asking about its size may have created a context in which the two measures appeared related or became confused with each other. This could occur because the dimensions graspable-to-ungraspable and small-to-big are conceptually linked. This could lead participants to estimate easily graspable objects as smaller, even if the visual representation of the object is unchanged. This possibility is supported by evidence from the literature on cross-sensory correspondences, whereby properties of one perceptual domain are linked to properties in another (e.g. Walker, 2012). For example, heavy objects are rated as darker than light objects (Walker, Scallon & Francis, 2016). ‘Graspability’ is not a perceptual feature like those studied in the cross-sensory correspondence literature. Nevertheless, a similar issue could have arisen in Experiment 2 of Linkenauger et al. (2011) if the experimental context implied a conceptual relationship between grasping capacity and object size. If so, then the results of Linkenauger et al. (2011, Experiment 2) could be explained by demand characteristics associated with performing two conceptually linked tasks on the same trial, as opposed to reflecting a change in what participants perceived in the strongest sense. Only the latter interpretation is consistent with the action-specific account.

We recently failed to replicate Experiment 2 of Linkenauger et al. (2011). In addition to testing for an effect of hand dominance, as was done in the original study, we directly manipulated grasping capacity by taping together the fingers of one hand (Collier & Lawson, 2017). This powerful manipulation restricted both actual (by ~1.2 cm) and perceived (by ~3.2 cm) grasping capacity. According to the action-specific account, taping should have influenced estimates of object size. However, although participants appropriately estimated the grasping capacity of their taped hand as less than that of their untaped hand, objects grasped in the taped hand were not estimated as larger than objects grasped in the untaped hand. We did not resolve why we failed to replicate Experiment 2 of Linkenauger et al. (2011), but we suggested that this could have been due to reduced context effects in our studies. This was achieved in two ways. First, in our initial experiments, participants completed the size estimation task before starting the grasping capacity task, so their size estimates were unlikely to be biased by considering the graspability of the objects. Second, the design of our final experiment was similar to that of Linkenauger et al. (2011, Experiment 2) in that participants were explicitly told that we were interested in their grasping behaviour, and the grasping task immediately preceded the size estimation task on each trial. However, our instructions emphasised that the grasping task and the size estimation task were part of two unrelated experiments.

In the present studies, we investigated whether we (Collier & Lawson, 2017) previously failed to replicate Experiment 2 of Linkenauger et al. (2011) because we reduced demand characteristics. In the present studies, participants had the fingers of one of their hands taped together, and we compared their estimates of object size for objects they had grasped in their taped versus their untaped hand. This taping manipulation has a number of advantages over the methods used by Linkenauger et al. (2011, Experiment 2). In their second experiment, Linkenauger et al. (2011) took advantage of the finding that right-handers perceive the grasping capacity of their right hand as greater than that of their left hand (Collier & Lawson, 2017; Linkenauger et al., 2011; Linkenauger, Witt, Stefanucci, Bakdash, & Proffitt, 2009). However, this only produces quite a small difference in *perceived* grasping capacity. Furthermore, there is no evidence for a difference in the *actual* grasping capacity of the right and left hands (Collier & Lawson, 2017; Linkenauger et al., 2011). In contrast, our taping manipulation alters both perceived and actual maximum grasp. In their final experiment, Linkenauger et al. (2011) manipulated perceived grasping capacity by magnifying the hand. However, as Linkenauger et al. (2011) themselves discuss (see also Witt, 2016), magnification could have induced a size-contrast illusion whereby objects may appear smaller next to a visually larger hand. It is therefore unclear whether the scaling effect they found in this experiment occurred because object size was scaled according to grasping capacity, or if it resulted from a size-contrast effect. In contrast, taping the hand directly reduces grasping capacity (Collier & Lawson, 2017) while minimising the possibility of inducing a size-contrast illusion (for a discussion, see Collier & Lawson, manuscript in preparation).

The action-specific account predicts that a change in grasping capacity due to taping the hand should influence perceived object size. Specifically, blocks grasped in the taped hand should be estimated as larger than blocks grasped in the untaped hand because the taped hand has a reduced grasping capacity. In Experiments 1 and 2, we tested whether previously reported effects of graspability on size estimates could instead be explained by *hypothesis guessing* by investigating whether participants were sensitive to demand characteristics arising from leading instructions. In Experiment 3, we examined the influence of demand characteristics due to *context effects* by having participants judge both how difficult a block was to grasp and its size on every trial. We expected that this would create a context which made grasping capacity seem relevant for estimating object size.

## Experiment 1

**Fig. 1:**
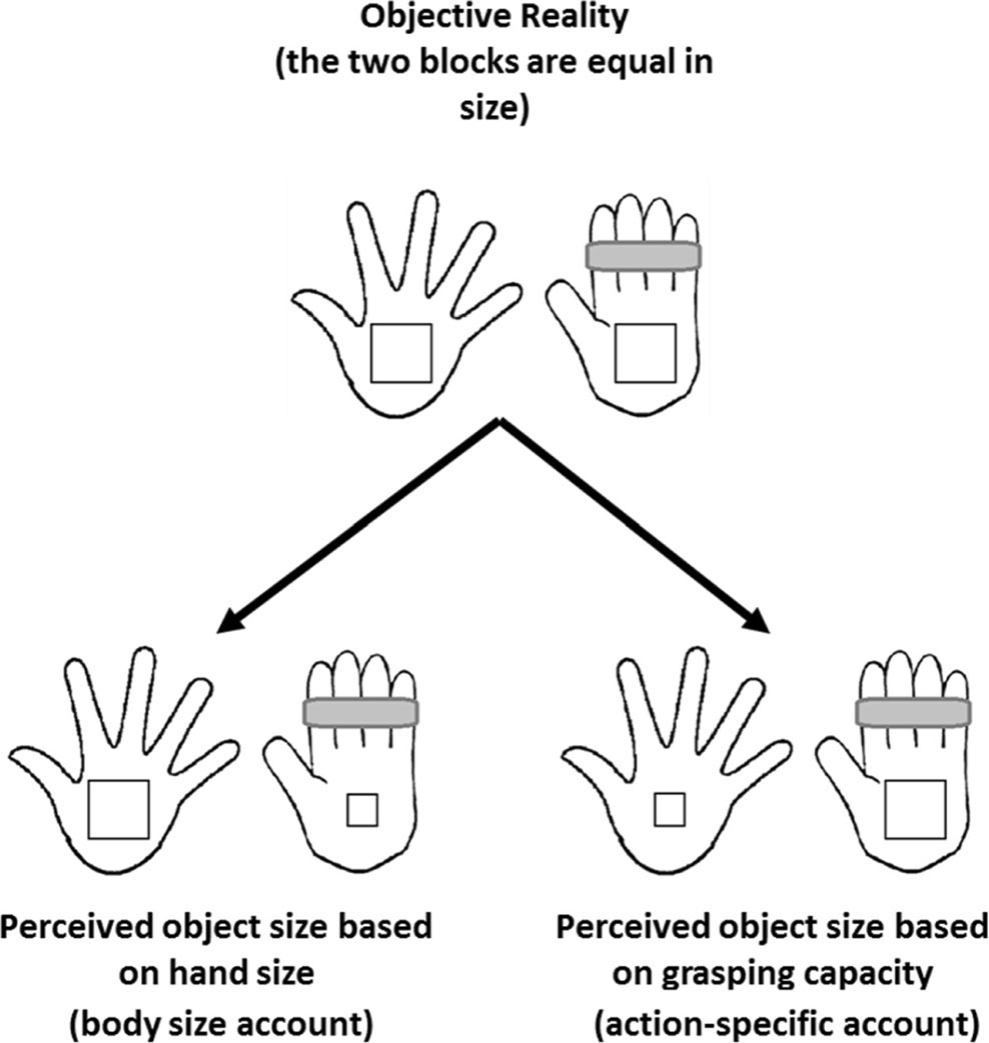
The predicted effects of instructions on perceived object size in Experiment 1. *Left:* Perceived object size *decreases* with a decrease in hand size due to taping (*body size account*). *Right:* Perceived object size *increases* with a decrease in perceived grasping capacity (*action-specific account*)

**Fig. 2:**
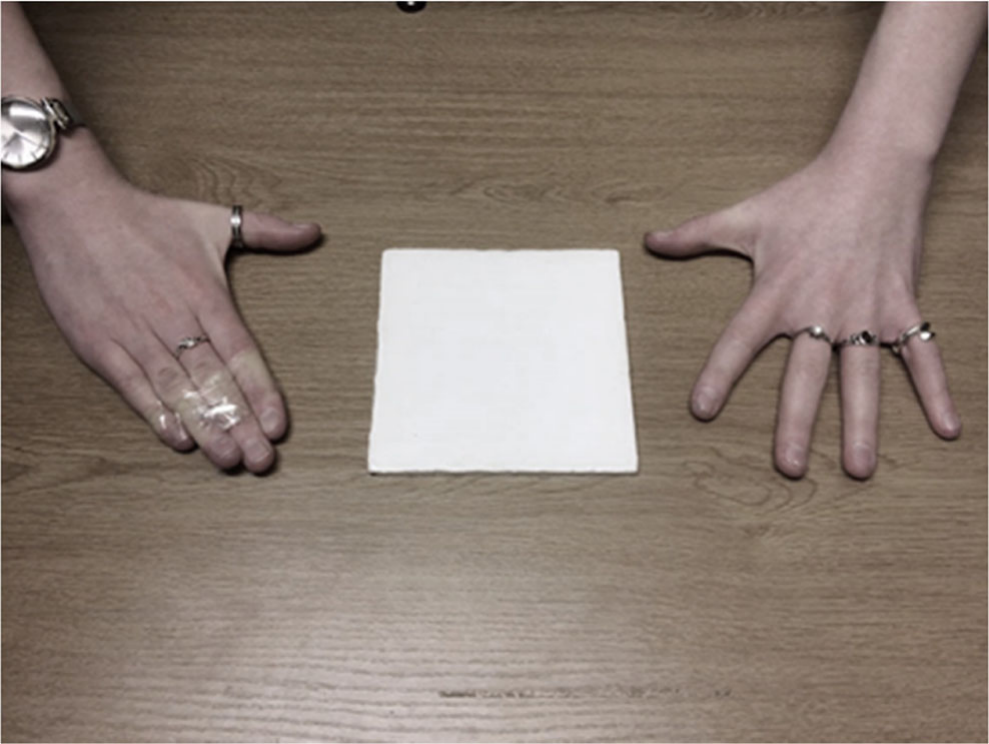
Photograph showing how the taping manipulation restricted the maximum grasp of one hand relative to the other. Image shows a participant in the RHTaped group following taping of their right hand. The hands are shown next to the largest (13 cm) block

Experiment 1 was designed to test whether participants would figure out the predicted influence of taping on estimated object size from the instructions they were given and then change their estimates accordingly. We reasoned that, depending on their instructions, hypothesis guessing could lead to two opposite effects (see Fig. 1). First, participants could be led to believe that objects grasped in their taped hand should look *larger* because taping reduces both the perceived and the actual maximum size of objects that can be grasped (Collier & Lawson, 2017). Here, hypothesis guessing would produce an effect in the direction predicted by the action-specific account. Alternatively, participants could be led to believe that objects seen near to their taped hand should look *smaller* because taping the hand makes it look smaller by reducing the maximum spread of the fingers (see Fig. 2), and because the taped hand could be used to anchor size estimates. In the latter case, using leading instructions which imply the opposite effect to that predicted by the action-specific account provides a strong way to test whether the effect reported in Experiment 2 by Linkenauger et al. (2011) was the result of hypothesis guessing. If participants are sensitive to leading instructions in this task, then they would be expected to comply with their instructions regardless of the outcome they imply. We therefore tested both alternatives. In the *action capacity group*, the instructions implied that objects grasped by the taped hand should appear larger because the grasping capacity of the taped hand is reduced, consistent with the action-specific account. In the *body-size group*, the instructions implied that objects near to the taped hand should appear smaller because that hand appears smaller, and this could cause the object to be scaled down in size. In the third, *objective-size group*, the instructions did not suggest that taping would influence size estimation, and participants were explicitly told to ignore nonvisual factors when estimating object size. Here, taping was not expected to influence object size estimates due to hypothesis guessing.

In Experiment 1 participants actually grasped each object they estimated the size of. In contrast, on each trial of Linkenauger et al. (2011, Experiment 2), participants only stated whether they thought they could grasp it. They did not grasp the blocks until the end of the experiment. Here, we tested actual grasping because we believe that the task used by Linkenauger et al. has low ecological validity. In everyday life, we often perform simple actions without explicitly attending to them (Goodale & Haffenden, 1998), whereas we rarely repeatedly decide whether we could act without actually acting. Also, action-specific scaling effects have been reported even when, as in our experiments, participants performed a relevant action without being explicitly asked if they could do it (e.g., Witt & Dorsch, 2009). Finally, Franchak and Adolph (2014) showed that participants only updated their perceived action capacity following a change to their body after they had actually performed the relevant action. This suggests that, for our taping manipulation to be effective, participants needed to try to grasp the objects with their taped hand.

In Experiment 1, we tested whether participants were sensitive to leading instructions which implied the desired experimental outcome. On each trial, participants first grasped and moved a block with either their taped hand or untaped hand, then placed that block next to a laptop. They then used the same hand to adjust the horizontal gap between two lines on the laptop screen to match the perceived width of the block they had just moved. If hypothesis guessing influences performance then we predicted that, relative to objects moved by the untaped hand, objects moved by the taped hand should be estimated as larger in the action capacity group, smaller in the body-size group and the same size in the objective-size group, see Fig. 1.

### Method

Ethical approval was granted for all of the experiments presented in this study by the relevant local ethics committee at the University of Liverpool.

#### Participants

Fifty-four participants (mean age = 18.7 years, seven males, *n* = 18 per group) were recruited for this study. Participants all self-reported as right-handed, and either volunteered or were rewarded with course credit for their time.

#### Design

Participants were allocated to one of three instruction groups (action capacity/objective size/body size). Throughout the experiment, participants had the fingers of one of their hands taped together. Half of the participants in each instruction group had their left hand taped (LHTaped group) and the remaining half had their right hand taped (RHTaped group). The middle and ring fingers were first taped together above the proximal interphalangeal (middle) finger joint, then all four fingers were taped together just underneath the same joint, see Fig. 2.

#### Apparatus, stimuli, and procedure

All participants received the following, general verbal instructions:

“In this experiment we will ask you to estimate the size of square stimuli. There are many possible interpretations of this instruction, so we want to make it clear what it is we want you to estimate. Imagine standing at one end of a road and looking at a house at the other end—the house may appear closer or farther away than it really is, depending on a variety of factors. For example, if you are very tired, hungry or in a rush, the distance to the house may appear greater than it really is. In contrast, if you are feeling very energetic, the distance to the house may appear shorter than it really is. These nonvisual factors have been previously suggested to influence spatial perception. The same logic applies to objects we can act on in our nearby environment. For example, if you are looking at a mug on a table, there may be things in the environment that make it visually appear closer to or further away than its actual physical distance from you (that is, the distance measured by a tape measure).”

Following this, they received group-specific instructions. The sentences highlighted in bold differed across the groups:

Action capacity group

“Similarly, this logic can be applied to the size of objects that we act on. For example, being able to grasp bigger objects may affect our perception of the size of objects we intend to grasp. In this experiment, we will tape together the fingers of one of your hands. This is to restrict the grasping capacity of one of your hands. You will then be presented with a series of square stimuli and asked to visually match their width on a screen. You will be asked to put either your left or right hand through the curtain to pick up the stimulus, take it out from behind the curtain, and place it on the table in front of you. Use the same hand you picked up the stimulus with to use the arrow keys to move the lines on the screen apart and visually match the width of the stimulus on the screen. **Base your answer on what size you feel the object is, taking all relevant nonvisual factors into account, including whether having your fingers taped together makes it harder for you to grasp big objects.**”

Body-size group

“Similarly, this logic can be applied to the size of objects that we act on. For example, thinking that our hand has decreased in size may affect our perception of the size of objects which we see near or hold in our hand. In this experiment, we will tape together the fingers of one of your hands. This is to simulate a shrinkage in the size of that hand. You will then be presented with a series of square stimuli and asked to visually match their width on a screen. You will be asked to put either your right or left hand through the curtain to pick up the stimulus, take it out from behind the curtain and place it on the table in front of you. Use the same hand you picked up the stimulus with to use the arrow keys to move the lines on the screen apart and visually match the width of the stimulus on the screen. **Base your answer on what size you feel the object is, taking all relevant nonvisual factors into account, including whether having your fingers taped together makes your hand feel smaller.**”

Objective-size group

“Similarly, this logic can be applied to the size of objects that we act on. However, if during this task you think that the objects appear to be different in size than how big you think they really are—for whatever reason—ignore these things and base your estimation only on how big you think the object really is. In this experiment, we will tape together the fingers of one of your hands. You will then be presented with a series of square stimuli and asked to visually match their width on a screen. You will be asked to put either your left or right hand through the curtain to pick up the stimulus, take it out from behind the curtain, and place it on the table in front of you. Use the same hand you picked up the stimulus with to use the arrow keys to move the lines on the screen apart and visually match the width of the stimulus on the screen.** Base your answer only on how big you think the object really is—imagine there’s a tape measure stretched across the object and you’re reading off its size.**”

After being given their instructions, participants completed a visual size-matching task. The stimuli were 10 foamboard blocks (0.5 cm thick). The blocks were square with sides ranging in size from 4 cm to 13 cm in 1-cm increments. In previous work (Collier & Lawson, 2017), this range was found to be graspable for most participants, even when their hand was taped. We only used graspable blocks because, according to the action-specific account, scaling effects are only expected if the relevant action is actually performable (Linkenauger et al., 2011).

**Fig. 3:**
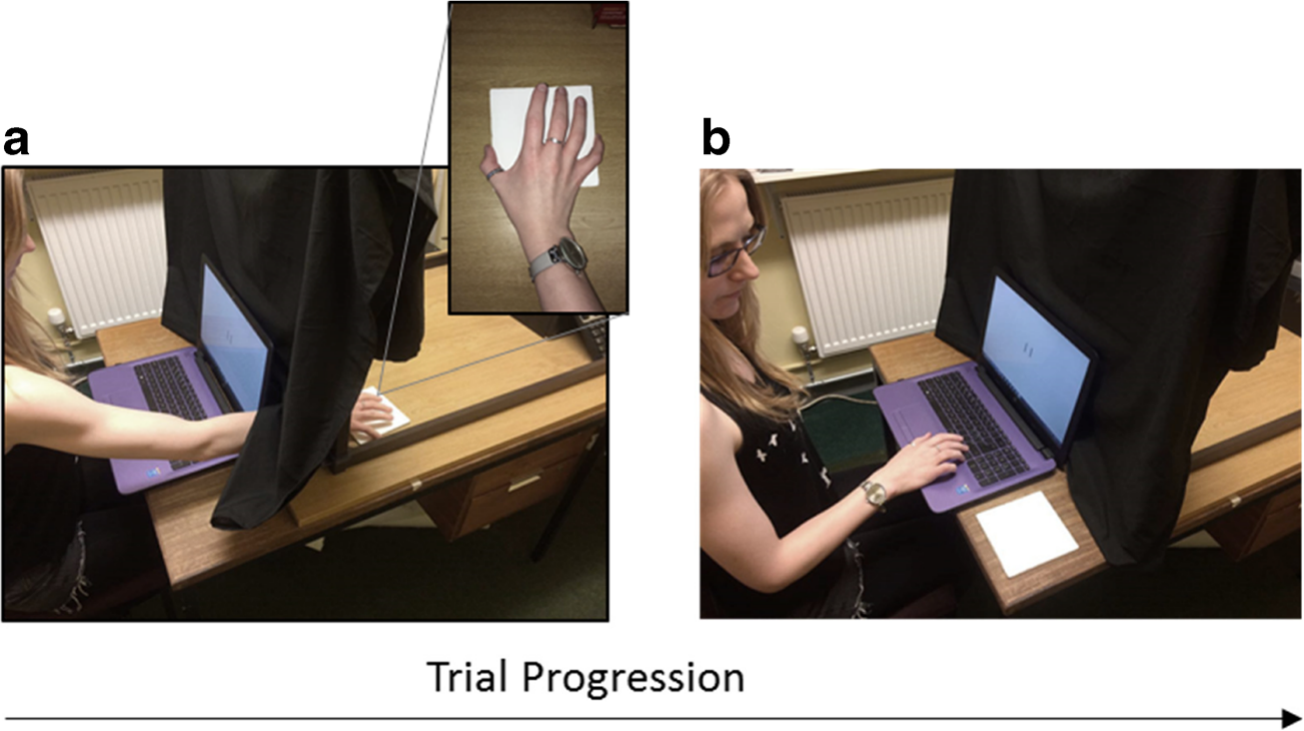
Trial procedure in Experiment 1 for an untaped right hand trial (the procedure was identical for the taped hand). **a** The participant has reached behind the curtain with their right hand to grasp and move the block (size shown here = 13 cm). The *inset* shows that the participant has successfully grasped the block using the specified grasp—the thumb on one side and any other finger on the opposite side. **b** The participant has moved the block to the right side of the laptop and placed it flat on the table. They are using their right hand to move the lines on the screen to visually match the width of the block. The experimental procedure was identical in Experiment 2. The experimental procedure was identical in Experiment 3, except that participants verbally rated how difficult the block had been to grasp before visually matching its size on the screen

On each trial, one block was presented on a table behind a curtain. A laptop (screen diagonal = 25 cm) was placed in front of the curtain. Two black lines (0.2 cm × 1.3 cm) were displayed on the screen. The lines were initially 0.9 cm apart. The participant reached behind the curtain to grasp and pick up the block (see Fig. 3a). The experimenter told the participant which hand they should use on each trial. The participant then moved the block onto the table in front of the curtain on the same side of the laptop as the hand they picked it up with (see Fig. 3b). Participants were instructed to always first try to grasp the block with their thumb on one side and any other finger on the opposing side (see Fig. 3a). If the block was too big to grasp in this way, they were then allowed to pick it up and move it in any way they wished. To maximise the likelihood of participants using the hand they had just acted with as a perceptual ruler, they pressed the response keys with the same hand they had just used to grasp the block and they kept their other hand out of sight, by their side. This ensured that they only saw the action-relevant hand while making their response. After responding, they used the same hand to place the block back behind the curtain. The experimenter then replaced the block with another block and the next trial began.

Before starting the experimental trials, all participants were given two practice trials which used the smallest (4 cm) and largest (13 cm) blocks. The 4-cm block was presented to their untaped hand, and the 13-cm block was presented to their taped hand. This was to try to highlight the difference in grasping capacity following taping. During the experimental trials, participants estimated the size of each block once for each hand, giving 20 experimental trials in total (10 blocks × 2 hands). Trials were presented in a different, random order for each participant. To minimise forgetting, participants were reminded of their group specific instructions after 10 trials. Specifically, the action capacity group was told to consider their grasping capacity, the body-size group was told to consider whether taping made their hand feel smaller, and the objective-size group was told to ignore all nonvisual factors while making their estimates.

After completing the size-estimation task, participants drew around their hands with their thumb and fingers spread as far apart as possible. They first drew around their taped hand (still taped), then their taped hand (with tape removed), and finally their untaped hand. They then completed a questionnaire on a computer. This asked what they believed the main manipulations of the experiment were, and whether they believed that their responses were influenced by having their fingers taped together and the experimental instructions. After this, the experimenter asked participants specifically whether they thought that having their fingers taped together had made objects appear bigger, smaller, or about the same size in their taped hand relative to their untaped hand. The entire procedure took about 20 minutes.

### Results

#### Object size estimation task

**Fig. 4:**
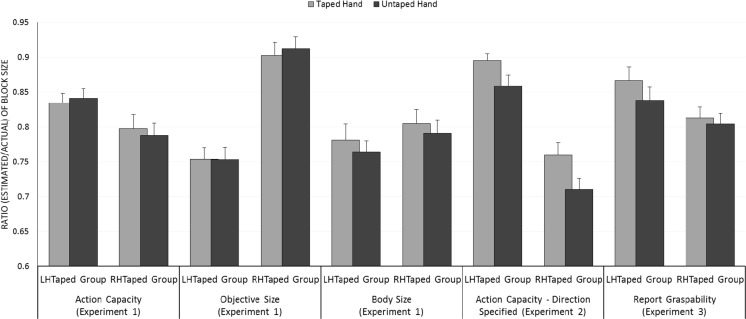
Mean estimated object size, shown as a proportion of actual object size, for the LHTaped and RHTaped groups in Experiments 1–3. *Error bars* show one standard error of the mean

We excluded six trials where the participant was unable to grasp the block in the manner specified using their taped hand (one 12-cm trial and five 13-cm trials) plus the six corresponding trials for that participant for their untaped hand. In addition, a further 16 trials were excluded due to invalid responses (e.g. pressing the Enter key without adjusting the distance between the lines). To test whether size estimates differed for taped versus untaped hands, we calculated perceived block size as a proportion of actual block size, then averaged these proportions for all block sizes tested for a given participant. These ratios were used as the dependent variable in a mixed ANOVA^1^ with Taping (taped/untaped) as a within-participants factor and Instruction Group (action capacity/objective size/body size) and Tape Group (LHTaped/RHTaped) as between-participants factors. There were no significant effects. For the main effects: Taping, *F*(1, 48) = 0.416, *p* = .5, η_p_^2^ = .01, Instruction Group, *F* (2, 48) = 0.754, *p* = .5, η_p_^2^ = .03, and Tape Group, *F*(1, 48) = 2.136, *p* = .2, η_p_^2^ = .04. For the interactions: Taping × Instruction Group, *F*(2, 48) = 0.517 , *p* = .5, η_p_^2^ = .02, Taping × Tape Group, *F*(1, 48) = 0.037, *p* = .8, η_p_^2^ = .001, Instruction Group × Tape Group, *F*(2, 48) =1.817, *p* =.2, η_p_^2^ =.07, and Taping × Instruction Group × Tape Group, *F*(2, 48) = 0.309, *p* = .7, η_p_^2^ = .01, see Fig. 4.

We also checked whether participants estimated block size in a way that was consistent with their beliefs about their own biases on this task, based on their postexperiment responses. To do this we analysed size estimates only for participants who chose the action-specific prediction (collapsing over instruction group and tape group, *n* = 26, see Table 4). If their post hoc beliefs were consistent with their experimental responses then they should have estimated blocks as larger for their taped hand. However, a paired-samples *t* test for this subgroup revealed no difference between their size estimates for their taped and untaped hand, *t*(25) = 1.419, *p* = .2.

We ran Bayesian analyses to test the strength of evidence for the null effects revealed by the ANOVA (see Table 1). We used the procedure described by Masson (2011), which determines the posterior probabilities for both the null and alternative hypothesis based on the Type III sum of squares values for the effect. This method can provide confidence that a null effect is not simply the result of a Type II error. We used the descriptive terms for strength of evidence suggested by Raftery (1995).

**Table 1:** Posterior probabilities for the null [p_BIC_(H_0_|D)] and alternative [(p_BIC_(H_1_|D)] hypotheses for the main effects and interactions in Experiment 1

Effect	p_BIC_(H_0_|D)	p_BIC_(H_1_|D)	η_p_^2^
Taping	.853**	.147	.01
Instruction group	.959***	.041	.03
Tape group	.694**	.306	.04
Taping × Instruction Group	.968***	.032	.02
Taping × Tape Group	.878**	.122	.001
Instruction Group × Tape Group	.883**	.117	.07

***strong evidence, **positive evidence

#### Hand span as an estimate of action capacity

We used participants’ drawings around their outspread fingers to estimate their maximum hand span to check whether this was reduced by taping. A mixed ANOVA was conducted with Hand (still-taped/was-taped-but-tape-removed/untaped) as a within-participants factor and Tape Group (LHTaped / RHTaped) as a between-participants factor. Hand was significant, *F*(2, 104) = 212.766, *p* < .001, η_p_^2^ = .80. Maximum hand span was lower for the still-taped hand than for either the hand that was taped but with tape removed or the untaped hand (see Table 2). There was no effect of Tape Group, *F*(1, 52) = 1.012, *p* = .3, η_p_^2^ = .02, or a Hand × Tape Group interaction, *F*(2, 104) = 0.026, *p* = .9, η_p_^2^ < .001. Thus the taping manipulation significantly reduced maximum hand span by ~4 cm regardless of which hand was taped.

**Table 2:** Mean (and standard deviation) of the maximum span of the still-taped hand, the taped hand without tape, and the untaped hand, in each group in Experiments 1, 2, and 3

	Action capacity group (Exp. 1, *n* = 18)	Objective-size group (Exp. 1, *n* = 18)	Body-size group (Exp. 1, *n* = 18)	Action capacity–direction specified group (Exp. 2, *n* = 18)	Report graspability group (Exp. 3, *n* = 18)	Grand mean (*n* = 90)
Taped hand (still taped, cm)	13.5 (1.8)	13.3 (1.9)	13.5 (1.7)	13.1 (1.6)	13.9 (1.9)	13.5 (1.8)
Taped hand (with tape removed, cm)	17.9 (1.2)	17.4 (1.8)	18.0 (1.3)	18.1 (1.2)	17.2 (1.8)	17.7 (1.5)
Untaped hand (cm)	17.6 (1.9)	17.0 (2.0)	17.9 (1.3)	17.6 (1.3)	17.5 (1.7)	17.5 (1.6)

#### Postexperiment questions

The number of participants across all groups in Experiments 1–3 who agreed that taping or instructions influenced their estimates of object size (Questions 6 and 8 in the questionnaire) is given in Table 3. The number of participants who responded that that objects appeared bigger, the same size, or smaller for trials using their taped relative to their untaped hand (asked verbally by the experimenter at the end of the experiment) is given in Table 4. Detailed responses to further open-ended questions can be found in the supplementary material.

**Table 3:** The number (and %) of participants in each group in Experiments 1, 2, and 3 who agreed in a postexperiment questionnaire that taping or instructions influenced their estimates of object size

	Action capacity group (Exp. 1, *n* = 18)	Objective Size group (Exp. 1, *n* = 18)	Body Size group (Exp. 1, *n* = 18)	Action Capacity - Direction Specified group (Exp. 2, *n* = 18)	Report Graspability group (Exp. 3, *n* = 18)	Total (*n* = 90)
Agreed that taping the hand influenced size estimates	15 (83%)	16 (89%)	14 (78%)	8 (44%)	14 (78%)	67 (74%)
Agreed that the instructions influenced size estimates	9 (50%)	14 (78%)	8 (44%)	15 (83%)	9 (50%)	55 (61%)

**Table 4:** The number (and %) of participants in each group in Experiments 1, 2, and 3 who agreed in a postexperiment questionnaire that objects appeared bigger, the same size, or smaller for trials using their taped relative to their untaped hand

	Action capacity group (Exp. 1, *n* = 18)	Objective-size group (Exp. 1, *n* = 18)	Body-size group (Exp. 1, *n* = 18)	Action capacity–direction specified group (Exp. 2, *n* = 18)	Report graspability group (Exp. 3, *n* = 18)	Total (*n* = 90)
Block appeared bigger in taped hand (action-specific prediction)	9 (50%)	8 (44%)	9 (50%)	11 (61%)	9 (50%)	46 (51%)
Block appeared no different in taped hand (objective-size prediction)	6 (33%)	8 (44%)	4 (22%)	7 (39%)	8 (44%)	33 (37%)
Block appeared smaller in taped hand (body-size prediction)	3 (17%)	2 (11%)	5 (28%)	0 (0%)	1 (6%)	11 (12%)

### Discussion

We did not find scaling effects on object size estimates as would be predicted by the action-specific account. In addition, estimates of object size did not differ between the taped and untaped hands in any of the three groups, so participants were not sensitive to leading instructions. We therefore found no evidence that differences in demand characteristics due to hypothesis guessing could explain why Collier and Lawson (2017) failed to replicate Linkenauger et al. (2011, Experiment 2). We re-examined this issue in Experiment 2.

## Experiment 2

In Experiment 1, the instructions given to the three groups may not have been sufficiently explicit to influence performance. For example, although the instructions for the action capacity group implied that grasping capacity might matter for size estimation, the expected direction of its effect still had to be inferred by participants. In Experiment 2, we investigated whether hypothesis guessing could influence performance if we directly told participants the results that we expected to obtain. We adapted the instructions from the action capacity group in Experiment 1 to explicitly tell participants that their estimates of object size were expected to be greater for their taped hand than for their untaped hand.

### Method

#### Participants

Eighteen participants (mean age = 18.5 years, zero male, mean Edinburgh Handedness Inventory score = 87.5, range: 50–100) were recruited for this study. Participants all self-reported as right-handed, and either volunteered or were rewarded with course credit for their time.

#### Apparatus, stimuli, and procedure

The apparatus, stimuli, and procedure were identical to that in Experiment 1, except for the following changes. First, participant’s fingers were taped before the instructions were read to them. This was to maximise the likelihood that, as they were given their instructions, participants would consider the relationship between grasping capacity and perceived object size that was being described to them. Second, only one set of instructions was used, which was adapted from the action capacity group of Experiment 1, as follows.

Action capacity–direction-specified group

“In this experiment we will ask you to estimate the size of square stimuli. There are many possible interpretations of this instruction, so we want to make it clear what it is we want you to estimate. Imagine looking at a mug on a table: There may be things in the environment that make it visually appear closer to or further away than its actual physical distance from you—that is, the distance measured by a tape measure. For example, if it appears difficult to reach, you may perceive the distance to the mug as greater than it really is.

Similarly, this logic can be applied to the size of objects that we act on. For example, the same thing might happen when we estimate the size of objects that we are going to pick up. In this experiment, we have taped together the fingers of one of your hands whilst your other hand has not been taped. Previous research has suggested that taping your hand makes it harder to pick up objects and that this makes objects grasped in or seen near to your taped hand appear bigger to you. Basically, because we are clumsier when our hand is taped, objects we might pick up with it appear larger to us so that we are more careful when picking them up.

In this experiment you will be asked to estimate the size of objects that you have just picked up with either your taped hand or your untaped hand. Take all relevant nonvisual factors into account when you estimate object size, including whether having your fingers taped together makes the objects appear bigger compared to your untaped hand.”

Participants were reminded that they should consider whether the blocks appeared larger in their taped hand after 10 trials, and the entire procedure lasted around 20 minutes.

### Results

#### Object-size estimation task

We excluded four trials due to invalid responses (e.g. pressing the Enter key without adjusting the distance between the lines). We calculated perceived block size ratios as in Experiment 1. These ratios were the dependent variable in a mixed ANOVA with Taping (taped/untaped) as a within-participants factor and Tape Group (LHTaped/RHTaped) as a between-participants factor. Taped hand estimates (*M* = 0.83, *SE* = 0.03) were greater than untaped hand estimates (*M* = 0.78, *SE* = 0.03), *F*(1, 16) = 7.282, *p* = . 016, η_p_^2^ = .31 (see Fig. 4). There was also a significant effect of Tape Group, *F*(1, 16) = 5.977, *p* = . 026, η_p_^2^ = .27, where LHTaped group estimates (*M* = 0.88, *SE* = 0.10) were greater than RHTaped group estimates (*M* = 0.73, *SE* = 0.16). There was no Taping × Tape Group interaction, *F*(1, 16) = 0.194, *p* = .7, η_p_^2^ = .01. As in Experiment 1, we ran Bayesian analyses to check the strength of evidence for the effects revealed by the ANOVA (see Table 5).

**Table 5:** Posterior probabilities for the null [p_BIC_(H_0_|D)] and alternative [(p_BIC_(H_1_|D)] hypotheses for the main effects and interaction in Experiment 2

Effect	p_BIC_(H_0_|D)	p_BIC_(H_1_|D)	η_p_^2^
Taping	.127	.873***	.31
Tape group	.196	.804**	.27
Taping × Tape Group	.792**	.208	.01

***strong evidence, **positive evidence

#### Hand span as an estimate of action capacity

We used participants’ drawings around their outspread fingers to estimate their maximum hand span to check whether this was reduced by taping. A mixed ANOVA was conducted with Hand (still-taped/was-taped-but-tape-removed/untaped) as a within-participants factor and Tape Group (LHTaped / RHTaped) as a between-participants factor. Hand was significant, *F*(2, 32) = 102.715, *p* < .001, η_p_^2^ = .87. Maximum hand span was lower for the still-taped hand than for either the hand that was taped but with tape removed or the untaped hand (see Table 2). There was no effect of Tape Group, *F*(1, 16) = 0.037, *p* = .9, η_p_^2^ = .002, or a Hand × Tape Group interaction, *F*(2, 32) = 2.912, *p* = .07, η_p_^2^ = .15. Thus the taping manipulation significantly reduced maximum hand span by ~4 cm regardless of which hand was taped.

### Discussion

In Experiment 2, participants estimated blocks as larger when they grasped them using their taped rather than their untaped hand. Thus when, unlike in Experiment 1, the desired outcome was clearly and explicitly stated in the pre-experimental instructions, participants produced scaling effects averaging ~4%. This effect is modest, but is comparable to the original effect reported in Experiment 2 of Linkenauger et al. (2011), where objects to be grasped in the right hand were estimated as ~3% smaller than objects to be grasped in the left hand. More generally, Firestone noted that many effects demonstrated by the action-specific account are only modest in size. He wrote that “paternalistic perceptual effects are the wrong size for the job” (Firestone, 2013, p. 458).

Our results are consistent with previous work suggesting that hypothesis guessing can influence performance to produce the effects reported in the action-specific literature (Durgin et al., 2012; Firestone & Scholl, 2014; Woods et al., 2009). The results of Experiments 1 and 2 suggest that in the size estimation task used both here and by Linkenauger et al. (2011, Experiment 2), participants may respond to demand characteristics from hypothesis guessing. However, this required instructions to be explict and overtly biased. Such extreme demand characteristics seem unlikely to explain the perceptual scaling effects reported in Experiment 2 of Linkenauger et al. (2011). In our final experiment we tried to resolve why scaling effects were obtained in Experiment 2 of Linkenauger et al. (2011) but not in Experiment 3 of Collier and Lawson (2017). We did this by examining the influence of a different type of demand characteristics on object size, namely *context effects*.

## Experiment 3

When action capacity and spatial properties are estimated in quick succession, and on every trial, as in Experiment 2 of Linkenauger et al. (2011), the experimental context may subtly imply that the two estimates are related, or the two types of estimates may become confused. Importantly, participants may not need to be aware of such context effects for them to occur, unlike explicit hypothesis guessing. Nevertheless, and importantly, scaling effects on spatial estimates arising from either type of demand characteristic are not genuine perceptual effects because the participant’s visual representation of the environment is not altered (Firestone, 2013).

On every trial in Experiment 2 of Linkenauger et al. (2011), participants estimated the graspability of an object immediately before estimating its apparent size. The dimensions of graspable-to-ungraspable and small-to-large may be conceptually linked, in a way similar to cross-sensory correspondences between sensory modalities (e.g. Walker, 2012; Walker et al., 2016). If so, then people may find it hard to assess them independently in a context where they are asked about both. We discussed this possibility in Collier and Lawson (2017) and referred to it as *conflation.* However, in our previous study, we did not test whether we could replicate Linkenauger et al.’s (2011, Experiment 2) scaling effect by introducing a context in which measures of spatial perception were likely to be combined or confused with estimates of action capacity. This was done in Experiment 3. Here, on every trial, participants rated how difficult the block had been to grasp (graspability) and then its size. Note that we were not interested in the results of the graspability task. The purpose of this task was to test whether drawing attention to the graspability of an object immediately before estimating its size would induce conflation between estimates of graspability and estimates of size. We reasoned that, in this conflation context, participants might estimate objects grasped in their taped hand as bigger than objects grasped in their untaped hand.

### Method

#### Participants

Eighteen participants (mean age = 19.6 years, two males) were recruited for this study. Participants all self-reported as right-handed and were rewarded with course credit for their time.

#### Apparatus, stimuli, and procedure

The apparatus, stimuli, and procedure were identical to those of the action capacity group in Experiment 1, apart from the following change. Participants completed an additional object graspability task on each trial. For this task, participants verbally rated the difficulty of grasping each block on a scale of 1 (*very easy*) to 10 (*very difficult*) after they had picked it up and placed it on the table. They then estimated the size of the block as in Experiment 1.

### Results

#### Object graspability task

We first tested whether participants rated blocks they had grasped in their taped hand as harder to grasp than blocks they had grasped in their untaped hand. Mean difficulty ratings were used as the dependent variable in a mixed ANOVA where Taping (taped/untaped) was a within-participants factor and Tape Group (LHTaped/RHTaped) was a between-participants factor. Participants rated objects they had grasped in their taped hand (*M* = 3.4, *SD* = 1.2) as more difficult to grasp than objects they had grasped in their untaped hand (*M* = 2.4, *SD* = 0.8), *F*(1, 16) = 21.519, *p* < .001, η_p_^2^ = .57. There was no effect of Tape Group, *F*(1, 16) = 0.814, *p* = .4, η_p_^2^ = .05, or a Taping × Tape Group interaction, *F*(1, 16) = 1.202, *p* = .3, η_p_^2^ = .07.

#### Object-size estimation task

We excluded two trials where the participant was unable to grasp the block in the manner specified using their taped hand (both were 13-cm trials) plus the two corresponding trials for that participant for their untaped hand. A further three trials were excluded due to invalid responses (e.g. pressing the Enter key without adjusting the distance between the lines). Perceived block size ratios were calculated as in Experiments 1 and 2. These ratios were the dependent variable in a mixed ANOVA with Taping (taped/untaped) as a within-participants factor and Tape Group (LHTaped/RHTaped) as a between-participants factor. Taped hand estimates (*M* = 0.84, *SE* = 0.03) were greater than untaped hand estimates (*M* = 0.82, *SE* = 0.03), *F*(1, 16) = 4.936, *p* = . 041, η_p_^2^ = .24. There was no effect of Tape Group, *F*(1, 16) = 0.771, *p* = . 4, η_p_^2^ = .05, or a Taping × Tape Group interaction, *F*(1, 16) =1.208, *p* = . 3, η_p_^2^ = .07 (see Fig. 4). As in Experiments 1 and 2, we ran Bayesian analyses to check the strength of evidence for the effects revealed by the ANOVA (see Table 6).

**Table 6:** Posterior probabilities for the null [p_BIC_(H_0_|D)] and alternative [(p_BIC_(H_1_|D)] hypotheses for the main effects and interaction in Experiment 3

Effect	p_BIC_(H_0_|D)	p_BIC_(H_1_|D)	η_p_^2^
Taping	.274	.726**	.24
Tape group	.735*	.265	.05
Taping × Tape Group	.688**	.312	.07

**positive evidence, *weak evidence

#### Hand span as an estimate of action capacity

We used participants’ drawings around their outspread fingers to estimate their maximum hand span to check whether this was reduced by taping. A mixed ANOVA was conducted with Hand (still-taped/was-taped-but-tape-removed/untaped) as a within-participants factor and Tape Group (LHTaped / RHTaped) as a between-participants factor. Hand was significant, *F*(2, 32) = 48.980, *p* < .001, η_p_^2^ = .75. Maximum hand span was lower for the still-taped hand than for either the hand that was taped but with tape removed or the untaped hand (see Table 2). There was no effect of Tape Group, *F*(1, 16) = 0.497, *p* = .5, η_p_^2^ = .03, or a Hand × Tape Group interaction, *F*(2, 32) = 0.596, *p* = .5, η_p_^2^ = .04. Thus, the taping manipulation significantly reduced maximum hand span by ~4 cm, regardless of which hand was taped.

### Discussion

In Experiment 3 participants rated objects as harder to grasp in their taped hand than in their untaped hand. They then went on to estimate blocks that they had grasped in their taped hand as larger than blocks they had grasped in their untaped hand. These results provide evidence for the suggestion by Collier and Lawson (2017) that the scaling effect reported by Linkenauger et al. (2011, Experiment 2) occurred because action capacity estimates were conflated with size estimates. This likely occurred because participants were asked to estimate graspability immediately before estimating object size on every trial. This influence of context would only need to occur occasionally to produce the modest scaling effects that have been observed (~3% in both Experiment 3 here and in Experiment 2 of Linkenauger et al., 2011). In Experiment 3 here, 11 out of 18 participants estimated blocks as larger for their taped hand than their untaped hand. Furthermore, participants appear able to independently estimate object graspability and size if care is taken to distinguish between them. For example, Collier and Lawson (2017) found that when participants were explicitly instructed that grasping and size estimates were being collected for separate, unrelated experiments, there was no influence of grasping capacity on estimated object size. Together, these results indicate that the scaling effect reported by Linkenauger et al. (2011, Experiment 2) was not truly perceptual.

## General discussion

In the present studies, we were interested in understanding the basis of biases that have previously been reported in the perception of object size and that have been interpreted as supporting the action-specific account. Specifically, Linkenauger et al. (2011) argued that apparent grasping capacity can influence perceived object size. However, we subsequently found no evidence to support this claim (Collier & Lawson, 2017). In the present studies, we sought to understand whether scaling effects were obtained by Linkenauger et al. (2011, Experiment 2), but not by Collier and Lawson (2017), because of differences in demand characteristics.

In Experiment 1, we investigated whether leading instructions would bias estimates of object size due to participants explicitly hypothesis guessing. We reasoned that estimated object size could increase if perceived hand size increased (on a body size scaling account), or could scale in the opposite direction based on changes in perceived grasping capacity (consistent with the action-specific account; see Fig. 1). Neither of these predictions were supported: We found no evidence that participants adjusted their responses after inferring the desired outcome of the experiment based on the instructions they were given. We re-examined this issue in Experiment 2 using a more powerful manipulation. Here, the instructions clearly and explicitly specified the direction of the expected effect based on the action-specific account. Now participants produced results consistent with the expectations arising from their instructions: Blocks that were harder to grasp because they were picked up in the taped hand were estimated as larger than blocks that had been grasped in the untaped hand. Taken together, these results suggest that hypothesis guessing is an unlikely explanation for the results of Linkenauger et al. (2011, Experiment 2) because scaling effects were only obtained in Experiment 2, when we used unrealistically directive instructions.

Orne (1962) stated that “response to the demand characteristics is not merely conscious compliance” (p. 779) and that other, subtler, forms of demand characteristics can also influence participants’ responses. Based on this suggestion, and our own proposal (Collier & Lawson, 2017) that conflation might explain Linkenauger et al.’s (2011, Experiment 2) results, in Experiment 3 we investigated whether the experimental context could implicitly influence performance. This was manipulated by having participants report an object’s graspability immediately before estimating its size. Now we found the predicted scaling effect: Participants estimated blocks as larger after grasping them with their taped relative to their untaped hand. This suggests that Linkenauger et al.’s (2011, Experiment 2) scaling effect likely arose as a result of asking participants to report graspability before object size on every trial. We propose that their task encouraged a conflation between estimates of action capacity and spatial extent, so that the scaling effects that they observed did not reflect a change in perception in the strong sense proposed by the action-specific account.

Our results expand on what is already known about demand characteristics in the action-specific literature by showing that these demand characteristics can take multiple forms. In Durgin et al. (2009) and Firestone and Scholl (2014), participants produced action-specific effects if no reason for a salient experimental manipulation was given, whereas participants who were given an explanation for the manipulation showed no effect. In these studies, action-specific effects seemed to occur only when participants guessed the experimental prediction. In contrast, the results of Experiment 1 here suggest that participants may not have explicitly guessed the experimental hypothesis in the object size-estimation task used by Linkenauger et al. (2011, Experiment 2). Nevertheless, the results of Experiment 3 here suggest that the scaling effect reported by Linkenauger et al. (2011, Experiment 2) could still reflect postperceptual demand characteristics due to an implicit context effect. Such context effects, like hypothesis guessing, are inconsistent with the explanation of scaling results provided by the action-specific account, namely, that participants actually see stimuli differently if their action capacity changes.

We previously demonstrated that context effects can be overridden using instructions which carefully distinguish between estimates of action capacity and estimates of spatial qualities. The final experiment reported in Collier and Lawson (2017) was similar to Experiment 3 here in that we asked participants to first grasp and then estimate the size of blocks on the same trial. Unlike Experiment 3 here, the experimenter emphasised that they were interested in participants’ grasping behaviour and said that they would record how participants grasped blocks on each trial. However, using a cover story about time constraints on data collection, participants were also told that the grasping task was producing data for a separate study to the size estimation task. In contrast to Experiment 3 here, we found no difference between size estimates made for objects grasped in taped compared to untaped hands in the final experiment of Collier and Lawson (2017). Thus, context effects were eliminated by telling participants that the tasks were separate, similar to the way in which hypothesis guessing was controlled for by Durgin et al. (2009), by giving participants a reason for wearing the backpack while estimating hill slant.

Thus previous work has found that providing a convincing cover story can eliminate action-specific scaling effects (Collier & Lawson, 2017; Durgin et al., 2009, 2012; Firestone & Scholl, 2014) and that the use of leading instructions can induce these scaling effects (Woods et al., 2009). In contrast, we found no evidence that explicit hypothesis guessing influenced estimated object size in Experiment 1 here. We suggest that this may have been because the experimental hypothesis was relatively hard to infer in this task, particularly since the group-specific instructions did not specify the direction of the predicted effect. Consistent with this interpretation, we did obtain scaling effects in Experiment 2, when participants were directly told the expected results of the study.

We have argued that scaling effects on estimates of object size may arise if these estimates are conflated with those of grasping capacity. Scaling effects were obtained in both Experiment 3 here and Experiment 2 of Linkenauger et al. (2011), when participants were *actively and explicitly encouraged* to think about and report their grasping capacity on every trial. It is important to emphasise that this context is unusual and does not reflect everyday life. Scaling effects were not obtained in the first four experiments reported by Collier and Lawson (2017), when participants were not encouraged to think about their grasping behaviour or capacity, even though they actually grasped blocks on every size estimation trial. Thus, scaling effects consistent with the action-specific account seem to be context-dependent, such that they only appear under narrow, non-ecological conditions.

Not all studies which have reported an influence of grasping capacity on estimated object size required participants to explicitly report their grasping capacity. For example, in Experiment 1 of Linkenauger et al. (2011), a disc was placed in the palm of the left and right hands of right-handed participants and they were asked which disk appeared larger. Participants also visually matched the size of the discs. In both tasks, the disks in the right hand were estimated as smaller than the disks in the left hand. Since participants did not have to report their grasping capacity, these results cannot be explained by context effects. There is though, an alternative explanation for these results which does not assume that action-specific scaling occurred. Right-handers have repeatedly been shown to believe that their right hand is larger than their left hand (Collier & Lawson, 2017; Linkenauger et al., 2009, 2011), so the discs surrounded by a perceptually larger object (the right hand) may have appeared smaller than the discs surrounded by a perceptually smaller object (the left hand). In fact, Linkenauger et al. (2011, Experiment 1) themselves suggested that such a size-contrast effect could have caused the results they obtained, rather than that perceived object size was scaled according to grasping capacity.

One reason that participants are asked to estimate their grasping capacity in studies supporting the action-specific account is to check perceived action capacity, since action-specific scaling effects are only predicted if people think that the action can be performed (Linkenauger et al., 2011; Witt, 2016). For example, only objects that people think they can grasp should be scaled; no effect should be found for objects larger than perceived maximum grasp (Linkenauger et al., 2011). One interesting issue, that has not yet been addressed, is whether scaling effects should be expected when objects are so small that they could be easily grasped regardless of whether they are grasped in the left or right hand, or indeed in a taped or untaped hand. Cañal-Bruland and van der Kamp (2015) suggested that distortions in spatial perception as a result of action capacity should be strongest at the critical boundaries for action. Investigating this hypothesis would be a valuable route for future research to pursue.

In order to produce a large, robust, yet reversible effect on both perceived and actual grasping capacity we used a taping manipulation in the experiments reported here. This differed from the manipulation of perceived grasping capacity investigated in Experiment 2 of Linkenauger et al. (2011). They took advantage of the bias for right handers to overestimate both the size and the grasping capacity of their right hand relative to their left hand. This bias existed prior to the start of the experiment and may arise from a lifetime of experience using their right hand more than their left hand. There is also greater representation for the right hand than the left hand in the somatosensory cortex of right-handers (Sörös et al., 1999). Such differences could be argued to explain why our results differed from those of Linkenauger et al. (2011). However, we think this is unlikely. First, Experiment 3 of Linkenauger et al. (2011) manipulated hand size by magnifying the hand. Like our taping manipulation, this is a short-term, within-experiment manipulation. Nevertheless, they reported differences in estimated object size when objects were placed next to the magnified, compared to the unmagnified, hand. Second, in our previous work, we found that participants rapidly updated their perceived grasping capacity after attempting to grasp objects with their taped hand (Collier & Lawson, 2017). This suggests that, although taping is a short-term manipulation, it is effective in influencing perceived grasping capacity. Thus, although our manipulation of grasping capacity differed to that used in Experiment 2 of Linkenauger et al. (2011), we believe our method is appropriate for investigating the effect they reported.

Modular theories of perception claim that perception is cognitively impenetrable, meaning that it is not affected by higher-level cognition (Firestone, 2013; Firestone & Scholl, 2015). The action-specific account challenges cognitive impenetrability by suggesting that perception can be directly influenced by action capacity. However, here we only found effects consistent with the action-specific account when the experimental instructions explicitly stated the expected outcome (consistent with hypothesis guessing), or when participants estimated object size in a context which implied that their grasping capacity was relevant (consistent with context effects). If apparent grasping capacity can directly influence perceived object size, as the action-specific account claims (e.g. Linkenauger et al., 2011), then we should also have found scaling effects when hypothesis guessing and context effects were controlled for (e.g. in Collier & Lawson, 2017), but we did not. The effects we observed in the present studies therefore seem to reflect biases at the level of judgement as opposed to true perceptual changes. By extension, our results are consistent with the idea of cognitive impenetrability.

In conclusion, the results of the present studies do not support the strong claim of the action-specific account that what we see is directly influenced by our action capacity. Our results instead suggest that the scaling effects on estimated object size that were interpreted as supporting the action-specific account by Linkenauger et al. (2011, Experiment 2) are more likely to have arisen from participants responding to subtle, easily overlooked cues within the experimental procedure. We are in agreement with Firestone and Scholl (2015) who observed: “If there is one unifying message running through our work on this topic, it is this: The details matter” (p. 59).

## Electronic supplementary material

Below is the link to the electronic supplementary material.

ESM 1 (DOCX 66 kb)

## Footnotes

1 For each experiment reported here we also tested for the original effect reported by Linkenauger et al. (2011) that objects grasped by the right hand would be estimated as smaller than those grasped by the left hand. In Experiment 1, a mixed ANOVA where grasping hand (left/right) was a within-participants factor and instruction group (action capacity/objective size/body size) and tape group (LHTaped/RHTaped) were between-subjects factors was conducted. There were no significant effects. For the main effects: grasping hand *F*(1, 48) = 0.037, *p* = .8, η_p_^2^ = .001, instruction group, *F*(2, 48) = 0.754, *p* = .5, η_p_^2^ = .03, and tape group, *F*(1, 48) = 2.136, *p* = .2, η_p_^2^ = .04. For the interactions: Grasping Hand × Instruction Group, *F*(2, 48) = 0.309, *p* = .7, η_p_^2^ = .01, Grasping Hand × Tape Group, *F*(1, 48) = 0.416, *p* = .5, η_p_^2^ = .01, Instruction Group × Tape Group, *F*(2, 48) = 1.817, *p* = .2, η_p_^2^ = .07, and Grasping Hand × Instruction Group × Tape Group, *F*(2, 48) = 0.517, *p* = .6, η_p_^2^ = .02. In Experiment 2, a mixed ANOVA where grasping hand (left/right) was a within-participants factor and tape group (LHTaped/RHTaped) was a between-subjects factor was conducted. There was no effect of grasping hand, *F*(1, 16) = 0.194, *p* =.6, η_p_^2^ = .01. There was a main effect of tape group, *F*(1, 16) = 5.977, *p* = .026, η_p_^2^ = .27; the LHTaped group had greater estimates (*M* = 0.88, *SE* = 0.10) than the RHTaped group (*M* = 0.73, *SE* = 0.16). There was also a significant Grasping Hand × Tape Group interaction, *F*(1, 16) = 7.282, *p* = .016, η_p_^2^ = .31. Bonferroni-corrected pairwise comparisons showed that for the RHTaped group, estimates were greater for the right hand than the left hand (mean difference = 0.05, *p* = .04), whereas there was no significant difference for the LHTaped group (mean difference = 0.04, *p* = .1). In Experiment 3, a mixed ANOVA where grasping hand (left/right) was a within-participants factor and tape group (LHTaped/RHTaped) was a between-subjects factor was conducted. There were no significant main effects: grasping hand, *F*(1, 16) = 1.208, *p* = .3, η_p_^2^ = .07, and tape group, *F*(1, 16) = 0.771, *p* = .4, η_p_^2^ = .05. There was a significant Grasping Hand × Tape Group interaction, *F*(1, 16) = 4.936, *p* = .041, η_p_^2^ = .24. Bonferroni-corrected pairwise comparisons showed that for the LHTaped group estimates for the left hand were greater than for the right hand (mean difference = 0.26, *p* = .032) but for the RHTaped group there was no difference between estimates for the left and right hands (mean difference = -0.009, *p* = .4).
