# Supplementary material for: Does grasping capacity influence object size estimates? It depends on the context
Source: Atten Percept Psychophys. 2017 Jun 21;79(7):2117–31. doi: 10.3758/s13414-017-1344-3 (PMC5603629; doi:10.3758/s13414-017-1344-3)
Supplement: Supplementary file 1 — (DOCX 66 kb) [file 13414_2017_1344_MOESM1_ESM.docx]

**SUPPLEMENTARY MATERIAL:**

**Comments made by participants in the online post-experiment questionnaire**

Here were report the responses made by participants in the online post-experiment questionnaire which were not reported in the main manuscript. Note that not all answers total to 18 because not all participants answered every qualitative question (quantitative questions reported in the main manuscript were forced-choice responses).

**Experiment 1: Action capacity group**

1. Please try to recall the instructions of this experiment in as much detail as you can, and write what you remember below. It is important that your description provides as much detail of the instructions as possible

- The ring and middle fingers will be taped together and then all four fingers will be taped on the left hand while the right hand will remain free. You will be asked to pick up blocks from behind the black curtain either with your left or right hand, and then using the arrow keys on the keyboard you will have to estimate the width of the blocks using the hand that you picked the blocks up with, taking into account any other external factors including whether or not the hand you were using is free.
- Will have one hand taped to restrict grasping ability. One hand will be free to grasp normally. Will be told which hand to use to reach though a cloth veil to grasp a square. Should then bring square onto the desk and using the same hand indicate the size of the shape on the screen. Two markers on the screen are controlled using the up and down arrows, up increases spacing, down decreases spacing. Then the squares should be replaced. Two practices will be performed followed by 2 sets of 10 'real' observations.
- Picking up the object behind the black cloth and judging its size by haptic perception and then recalling what we thought the size of the object by using the arrows on the keyboard to move the two lines appropriately to match the width of the object.
- One of my hands were going to be taped together and we had to alternate between different hands when reaching through the gap for the different sized shapes. We then had to move the two lines apart until we thought they were the same size. It was based on if our hands felt different sizes or not.
- Firstly I had my right hand cellotaped together. My two fingers in the middle were cellotaped first and then all 4 of my fingers were cellotaped. I then had a trial run of what to do in the experiment. I then had to pick up square objects from under a black cloth with either my left or right hand and move the arrows on the computer to what I thought the width of the shapes were. After 20 of these I had to draw around my hands with black pen on a sheet. I then had to draw over the cellotaped hand with my hand without cellotape on it in red pen.
- You will be given different sized objects to pick up, randomly alternating your left and right hand, with one of your hands being taped up. You will be asked to estimate the distance by using the up and down key and you must take into consideration the difficulty of picking up the object
- To pick up the objects with either the right or left hand and then try to match the size with the same hand using the 2 lines on the screen.
- I was asked to use either my right hand (which was taped together) or my left hand to pick up a square either large or small through a black curtain once I had picked up the square I had to estimate the width of the square using the computer.
- To pick up the shapes with the hand I am told to, and to see if the taped hand makes it harder to estimate the size of the shape.
- My right hand was taped up then I was shown two vertical lines next to each other. I was told to put my left or right hand through a curtain to pick up different sized squares. Then I had to increase the distance of the lines to fit the size of each square.
- I have my fingers on one hand taped together and must pick up objects with the specified hand and attempt to judge how big they are by moving lines on a screen, using the hand that I had to pick up the object with.
- 2 lots of 10 trials. Each trial, reach through the curtain with whichever hand is instructed by the experimenter, and move the lines on the screen apart the same width you feel the object it, using the hand you were instructed to reach with. My right hand was taped, and my left hand wasn't.
- I was instructed to use either my left or right hand (which was taped up) to collect square stimuli from behind a black curtain. I then had to use the same hand as I picked the object up with to move the arrows on the computer in order to make the distance between two lines the same as the width i thought the square stimulus was.
- I was told after taping my right hand's fingers together that I would be asked to pick up the squares underneath the curtain with either my left or right hand and then using that same hand get the two lines on the computer screen to match the width of the square I had just picked up. The same hand that picked up the object was the hand used to estimate the width of the square on the screen.
- To bear in mind how having taped fingers on my left hand affected my estimation of the width of the objects. To pick up the object using the hand instructed to use and then with the same hand to estimate the width of it onto the computer screen.
- We were asked to tape our right hand up and then we would be told which hand to use (left or right) to pick up a flat square shape of different sizes through a small black curtain by using the full width of our hand. We then used the up and down buttons on the keyboard to estimate the size of the square (making sure to use the same hand as the one we picked the shape up with) and take into consideration how easy it was to pick up the object when estimating. We then used that hand to put the object back behind the curtain and repeat the process using different hands. There were two sets of ten trials.
- Try to estimate and match the size of a square taking into account all external cues which may bias the estimation

1. What do you think the overall aim of the experiment was?

- To investigate the effect of external factors such as whether the fingers were taped or not on how we perceive objects i.e. the ability to accurately estimate the width of blocks.
- To explore the relationship between perceived distance and actual distance with restricted and unrestricted grasping capabilities with each hand.
- Whether we can use our haptic perception to appropriately and accurately match the size of objects
- To see if the way we judge how big a distance is if affected by different factors.
- To test the effects of estimating object sizes depending on whether you had use of all fingers or not
- To see if taping the hand had an affect on the judgement of size.
- To see if using your left hand made a difference to your estimate if you are right handed
- To see how people's awareness of space is affected by limited movement of the hands (one of the senses).
- To see if having one of your hands taped up made it more difficult to estimate shape size
- To see if perception is changed by which hand you pick something up with
- To assess whether environmental differences effect our ability to estimate the size of objects.
- To test how participants perception of objects change due to different conditions.
- To see whether there is a link between estimation of shape width and perception is affected when senses (in this case my sense of touch and general perception) are limited.
- To measure how the estimation of objects may vary depending on the hand used (taped or not taped)
- To find out if my perception of length is altered by which side of me something is? Still not sure what the finger tape was for!
- To see if people over or under estimate the size of objects depending on how hard it is for the person to pick the object up.
- Touching a set of pvc squares

1. What do you think the main manipulation of the experiment was?

- The taping of the fingers on the left hand.
- Taping fingers together to restrict grasping ability.
- The size of the object and hands; one taped one not taped
- Having one hand taped and one had not.
- Cellotaping your right hand
- The participants hand being strapped up or not
- The taping of the hand.
- The right hand being taped together
- The taped hand
- Having one hand taped up and comparing the difference between the taped up hand performance and the non taped up hand performance
- That one hand was taped together, whilst another could more easily get a feel for the size of the object
- Whether your hand is taped or not, and which hand is taped
- Having the fingers of my right hand taped together.
- To see if the participants were affected when their main hand was hindered and limited to doing the task
- The taped fingers- this may have been used to measure the difference that each condition makes in estimations
- Right or left hand
- Whether you were picking the object up with a hand taped together or not.
- Having fingers of one my hands stuck together

5. What did you think was specific in your instructions? Did anything stand out to you?

- The instructions were very clear and thorough, emphasis was made on taking into account all external factors.
- To focus on the tactile/haptic feedback more so than the visual. To use the specified hand for both manipulating the object and for giving feedback.
- Must use the same hand that you picked the object up with to move the lines
- The way in which they were given were clear and were fully understandable
- That I had to pick up objects from under a black cloth and measure their width
- To take into account the difficulty of picking up the object
- Having to use the same hand to move the lines on the screen.
- No the instructions were very clear
- To see if the taped hand felt differently and made it harder
- That I had to use the same hand to adjust the length as the one I picked the shape up
- The fact that the hand which you used to pick up the object had to be the hand which you used to adjust the lines with
- Which hand to reach through the curtain and to operate the computer
- That I had to pick up the object with the same hand that I then had to use to estimate the size of the object.
- Having to use the same hand on the screen as the one that picked up the object.
- Having to bear in mind how having taped fingers may affect my estimation
- To ignore the other factors influencing what could change my perception. And also that I need to try and imagine the tape.
- The fact that we had to take into consideration how easy it was to pick the object up when estimating its size
- I was told exactly what to do and to which aspects of the task I should pay attention to

7. What effect do you think the instructions had on your behaviour or responses? Please be as specific as you can, including the direction of any predicted effects.

- I tried to focus on the difference in how I feel when picking up the blocks with my left compared to my right hand. However, I feel as though there was not much difference for me to estimate the blocks differently.
- Tried to focus on the stimulus from my hands and not so much from my eyes. I am kinda wondering why I got to see the square though. I expect I will be overthinking the responses I give from my taped right hand, hence a longer response time for that hand.
- Using more concentration when moving the lines as I attempted to use my dominant hand to move the lines
- Made sure that we were basing our responses on how big or small our hands felt.
- If the researcher asked me to use my right/left hand then I would pick up the square with that hand
- I found it harder to pick up the shapes but didn't feel that having my hand taped affected my ability to estimate the size of the different shapes
- They ensured that I listened to which hand I had to pick up the object with and made sure that I used this hand to adjust the lines, as it is likely I would have used my favoured right hand to adjust every line
- I don’t feel it had an effect on my behaviour
- I think the instructions made me pay more attention to what I was doing in order to not make any mistakes in terms of using a different hand to the one I was instructed to use. I was definitely more particular in predicting the size of objects using my right hand because I think thats the hand I’m predicted to do worse in as my fingers were taped together.
- Knowing that the aim was to see if taping the hand gave more error in guessing the width may have made me more aware that it was harder to estimate when using my right rather than my left hand
- I think being told to bear in mind how having taped fingers affected my estimation of the width of the shapes made me more aware of it, so I started to compare how much time I tended to take in estimating the width of an object with taped fingers and then non-taped fingers
- I think that I was more aware of potential differences in my predictions when using either hand
- I think that when I picked up an object with my right hand (the taped hand) I was slightly overestimating the size of the object, because it felt bigger as it was harder to pick up.
- Although I followed the instructions I don't think they affected the way I estimated the sizes of sqrs

9. What effect do you think that taping your hand had on your behaviour or responses? Please be as specific as you can, including the direction of any predicted effects.

- Trying to figure out the purpose of taping my hand as it doesn't seem like something that could have a big influence on the perception of the width of blocks.
- It altered the way I picked up the object. Made me use my index finger and not my first finger to give responses on the tablet. Overthinking might have lead to overcompensation, e.g. if I cant grasp it I might think its bigger hence I might over compensate by estimating a smaller size.
- By taping my hand together my hand felt a lot smaller and so this probably made the distance of the object bigger.
- It was more difficult to pick up, therefore it made me estimate the object as larger than with my left hand
- I don't think it affected my responses
- Made my estimates smaller
- I could not fully grasp the object and did not get a feel for the object, as it was a lot more difficult to pick up
- I found it more difficult to predict the size of the object when my hand was taped as there was no comparison with my hand span
- I think I did better in predicting the objects size with my taped hand because I had to concentrate more.
- It was more difficult to guess the width of the square on the screen when using my taped hand, especially with the larger squares as it was more difficult to pick them up and determine their size.
- I feel that maybe the unusual feeling or discomfort of it may have caused me to quickly estimate the width of objects when using my left hand (taped), compared to using my right hand (not taped)
- I wasnt aware of any affect it had on my behaviour
- Again I found the objects harder to pick up so I think I over estimated the size rather than under estimating it
- I don't think my responses were influenced by my hand being taped

10. Having answered these questions, what do you think the overall aim of the experiment was?

- Whether factors such as detailed instructions and the taping of the hand have an effect on estimating the width of blocks. I am guessing in other conditions the participants would have both of their hands free/receive less detailed instructions.
- To actually look at how peoples ability to guage lengths changes with their ability to grasp something. Then to examine how I interpreted the instructions, and assess how much I was thinking about what I was doing.
- Whether the taping of one hand affected your haptic perception
- To see if factors for different people affect the way in which we judge distance.
- To see whether having your hand cellotaped will affect your visual perception of objects
- To look at the effects of certain factors on estimating object size
- To see if taping the hand affected the judgement of the size of the object.
- I think the overall aim was to test if right handed participants can estimate objects sizes better using their left hand because the right hand was taped
- To see if being told to look if it affected the response, the response would be poorer than with the other untaped hand
- To see the effect of having a hand that is incapable of grasping on guessing shape sizes
- To see what factors attribute to perception of size
- To see how taping a hand and the instructions given,effects how people estimate the size of objects
- To test whether having one hand taped together would improve my performance of estimating the size of the objects.
- To see if estimating the size of objects is influenced by mood or senses being limited.
- To measure how feeling/ mood may affect the time taken when completing a task
- To see if my perception of length is altered by which hand I used, or which side I had to put the shape on
- If people estimated the size of objects differently depending on if they found the object hard to pick up or not.
- To make the World a better place

**Experiment 1: Objective size (control) group**

1. Please try to recall the instructions of this experiment in as much detail as you can, and write what you remember below. It is important that your description provides as much detail of the instructions as possible

- I was instructed to estimate the true size of each square, reaching for each square with a different hand and inputting the size with that hand only
- Using which hand I am told to use, reach in front of me behind the black curtain and grab the square block with my thumb on one side and a finger on the other and put it next to me. Using the same hand I grabbed the block with, press the up or down arrow to adjust the two lines on the screen to match the width of the square next to me, then repeat this process many times. Following this I had to draw round both my hands, then remove the tape from my right hand and draw round it with my fingers spread.
- With my left hand taped (ring and middle finger first, then all four fingers) and my right hand free I had to take a square shape from behind a screen using the hand I was asked to use. I then had to use that same hand to alter the size of two lines to show the width I thought the square was, without allowing any other factors to influence me.
- When I am told a side, I place that hand through the small curtain infront of me and pick up the "square" behind it and place it down on the same side then with the same hand I use the arrow keys on the computer infront of me to adjust the bars on the screen until they match the same distance apart as the stimulus is wide. I press enter once this has been completed and put the stimulus back through the curtain with the same hand. This repeats 10 times with different hands.
- You are going to be measuring the width of a selection of square objects, you will have one hand taped and you will be asked to place either your left or right hand through a curtain. There will be lines on a computer and you have to move them to represent the width of the square, taking into account the difficulty of when picking them up.
- Fill out a consent form, stick left hand fingers together with tape, pick up each object with the hand told to, estimate how wide each object is by moving the up and down key
- Having the left hand taped, had to put left or right hand through a curtain to pick up a shape. Using the up and down keys on the laptop there was two lines that I could make bigger or smaller to objectively measure the size I percieved the shape. The hand picking up the square would be the hand to be used when measuring the size on the computer screen.
- Attempt to visualise and estimate the size of the object without any other feelings based upon your decision, just what size estimate that you believe it to be as if you are measuring with a tape measure.
- In this experiment I was asked to estimate the size of a square. My left hand was taped at the beginning of the experiment. The researcher passed me some squares under a black curtain. If I used my right hand to pick the square then I should use my right hand to type on the keyboard in order to estimate the size. If I used my left hand, the process was the same but it was the left hand that typed. There were 2 moving lines on the computer screen which was used to estimate the size. I was told that the size I estimated should be the size I thought it was.
- At first the researcher tied my right hand. She passed a square object to me and I collected it using only one hand to estimate the width of it and than try to make the same width on the screen. Finally, pass it back. Repeat this process again and again.
- Experimenter will either say left or right, use that hand to reach through the black curtain and take the object and put it in front of me and use the arrows on the keyboard to move the lines on the screen, using that hand to estimate the width of that object imagining the object is on a tape measure and I'm reading off the tape measure.
- Take the square block of the stimuli by using taped hand and left hand and try to adjust the length between two lines on the computer screen until it matches the width of square block.
- I had my left hand taped. I was told to reach through a covered area and pick up a square with either my right or left hand then move the lines accordingly, using the same hand I picked the shape up with to press the arrow key to move the lines. I was asked to move the lines so that they were the same distance apart as the square was wide and to pretend that the square had a measuring tape across it in order to gage size.
- Have to use the arrow keys to estimate the size of the white tile as accurately as possible, as if it was being measured with a tape measure
- One hand will have its fingers taped together. You must put your hand through the curtain with the hand you are given e.g left or right and estimate the size of the square you pick up. Whilst doing this on the screen you must use the same hand you picked up the object with trying to estimate the size as accurately as possible.
- The researcher either asks you to take the object behind the black cloth with your right or left hand. Then with whichever hand you took the object with, you need to either press the "up" or "down" keys to manipulate the length of the lines to match the physical width of the object. The width should equal the same as placing a measuring tape on the object.
- I was told that my right hand fingers would be taped together then I was told that I would have to remove a square shape from behind the curtain with either my left or right hand at random then asked to measure the width of the square as if there was a measuring tape across it. I the had to use a computer program to estimate the width of the square using arrows with the hand I used to retrieve the square
- I will get two sets of 10 line tasks and I will have my right hand taped up. I will have to pick up an object with either my taped right hand or my free left hand and with that same hand, I will have to try estimating the width of the square. After this, I will first have to draw around my taped right hand with a green pen. Then I will have to draw around my free left hand with my fingers open as wide as possible. Next I will have to take off the tape and draw my right hand with my fingers wide apart.

1. What do you think the overall aim of the experiment was?

- Not sure
- To understand whether perception of width is affected by the way we hold the object
- To see if people can show the width of something correctly without actually measuring it and allowing other factors to influence it. Testing our perception of reality?
- To convince my mind that a visual/haptic distance is further than it actually is by making the data available to my brain harder to come by: hence the tape.
- To look at how difficulty in picking up an objects affects perception of the width.
- To see if having your fingers closer together changes the way you estimate the width of objects
- To see if perception of size is objective and accurrate
- To see if your dominant hand is more effective at estimating the size of objects.
- The aim is to explore our perception on the size of certain objects based on different hands. If the participant is a right-handed, was his/her left hand perception more or less accurate than the other hand?
- To measure the ability of estimating the width in both hand-tied or not condition.
- To see if factors such as having one hand taped up affects our perception of how big objects are.
- It tried to study how visual reception of the of the stimuli affects the reception of the information of the brain
- To see if participants would guess the square to be wider when using the impaired hand (taped) in comparison to when the normal hand was used.
- To see if inability to grasp objects properly affected size perception?
- To see how not being able to use one hand properly may affect the way you estimate the size of objects?
- To see whether a participants judgement of the physical width of an object can be altered depending on if fingers are closer or further away.
- I think the aim could be to see whether the right and left handed estimates would differ to see the dominant hand.
- I think it was to see whether we would estimate the width of the box at a larger or smaller size depending on the size of the box we got previously.

1. What do you think the main manipulation of the experiment was?

- Not sure
- Whether the hand used to pick up the block was taped together or not
- Having my fingers taped and that I had to use the same hand I picked the square up with to alter the width on the screen.
- Having a hand taped versus having a free hand.
- Difficulty in picking up the object. Having fingers taped together
- Which hand was used to pick up the square.
- Taping up the fingers on the right hand.
- The use of left hand or right hand.
- The width of the objects
- Having my non dominant hand taped up.
- It limits the ability of the right hand because it is taped and cannot be used as usual.
- Whether the hand was taped or not.
- Taped hand
- Having fingers taped together.
- Whether the fingers are taped together or able to move freely
- The left and tape on the right hand
- The order of the size of boxes given.

5. What did you think was specific in your instructions? Did anything stand out to you?

- That I had to tape one hand and not the other
- The way I was told to pick up the blocks with my thumb on one side and a finger on the other side and use the same hand to touch the arrows
- Using the same hand to pick the card up as to alter the width of the lines on the screen. And also not to look underneath them.
- The fact that I must use the same hand for every action in a trial. No, this seemed standard.
- Being told which hand to use to pick each object up with
- To always be objective and use the same hand on the keys which picked up the square
- To visualise the tape measure
- The researcher demonstrated some trials to me on how the experiment should process at the beginning of the experiment, that was quite straightforward and clear.
- No
- Specifically kept reminding me to imagine there was a tape measure on the object and that I was reading from the tape measure.
- Doing adjustment with the same hand which takes out the objects
- To imagine there was a tape measure across the square. To use the same hand to type.
- Describing the house and the effects of mood on visualisation of distance
- The emphasis put on needing to type/change the space size with the hand you were told to pick the object up with.
- I could only tap the keys on the keyboard with the hand I picked the objects with
- The instruction of how to retrieve the square from the curtain and measuring the width of the squares using the same retrieval hand to estimate the size of the square on the screen
- Giving a backstory using examples of houses and distance helped to understand the instructions better

7. What effect do you think the instructions had on your behaviour or responses? Please be as specific as you can, including the direction of any predicted effects.

- The instructions were making the experiment seem a lot more complicated than it was.
- I picked up the blocks as instructed to do so
- I was more conscious of getting the width correct I was worried I was getting it wrong each time.
- I ensured I was definitely using the hand instructed at the start of each trial.
- Not sure
- Made me feel like I should be honest and accurate on the perception if the square, I would try and guess which hand would be up next as I was told it was randomly allocated but seemed to be the most manipulated
- To be more doubtful about the answer given and attempt to be as accurate as possible.
- No much effect. Just introduce how the experiment processing.
- The instruction mentioned in previous question. It affects me that sometimes I will do the adjustment by using right hand but I took out the object by using left hand.
- I may have guessed the objects picked up with my left hand to be bigger than the ones picked up by my right.
- I don't think they had any major influence?
- The clear instructions made me slightly more focussed and made me make sure I concentrated a little more
- I followed the instructions
- The instructions didn’t influence behaviour too much because there was so much detail in them that it was harder to guess what the experiment was actually about (assumming there was some deception involved).

9. What effect do you think that taping your hand had on your behaviour or responses? Please be as specific as you can, including the direction of any predicted effects

- I think I didn't perceive the width of the block as accurately when using my taped hand to pick up the block
- I'm only thinking now that maybe holding it for longer could have helped me determine it's shape as at the time I preferred to determine it's width just by looking at it. Taping my hand didn't really bother me physically except for the fact I couldn't pick the larger pieces up.
- I did not think that taping my hand influenced my behaviour or responses.
- Felt that I was more accurate, as it was harder to pick up so I could feel the objects surface more.
- It made the shape harder to pick up
- Made me feel like I lacked in control making it harder to concentrate on the size of the square
- It restrained the touch of the object when picking it up and when clicking on the arrows to guess the size.
- Taping my hand makes it easier for me to estimate the width because it makes my hand fixed.
- It was harder to estimate the size of the objects as it was harder to pick up the objects so I couldn't estimate the size using my hands I just had to use my eyes.
- It is difficult to take the object out.
- Taping my hand may have impaired my ability to perceive object size.
- My right hand is my dominant hand so it was awkward not being able to use it properly
- I think taping my hand would have impacted my estimation of the shapes I picked up with the taped hand, I may have seen them as being bigger as they were harder to pick up.
- I thought the width of the object was bigger than in reality
- I couldn't hold the square as easily
- I don't think it had much of an effect on behaviour because it still felt the same picking up the square with either hand.

10. Having answered these questions, what do you think the overall aim of the experiment was?

- To see if the instructions given changed our perception of items.
- To understand the effect that taping someone's hand has on the way they perceive the width of the blocks they pick up using that hand
- To determine whether we judge somethings size using our touch as well as eyesight.
- The same thing I thought it was the last time I was asked that.
- To understand how the difficulty of the task influences perception of the width of objects.
- Too see if taping your hands together changed the way you estimated the size of shapes
- If perception of size changes in unusual circumstances
- To see if touch can help estimate the size of object, rather than just relying on sight
- The aim is to estimate the the size of certain objects. Will our perception be influenced by the left hand use or the right hand use?
- The instructions and taping hand’s influence in the people's ability of estimating width of objects.
- To see if specific instructions affect the way the participant perceives the size of objects.
- It tried to understand the difference between using left hand and right hand when we are asked to take out or hold the object. Also, it also tried to understand how visual reception of the stimuli affects how brain receive the information.
- To see if an impairment causes size estimation to differ.
- I have no idea
- To see if there is a link between distorted estimation of sizes and lacking the ability to use one hand due to the fact the objects would be harder to pick up, therefore appearing bigger.
- To see if the participants judgement of the objects width can be altered depending on the hand they picked it with
- I still think its about the order of the size of boxes influencing how you would estimate the width.

**Experiment 1: Body size group**

1. Please try to recall the instructions of this experiment in as much detail as you can, and write what you remember below. It is important that your description provides as much detail of the instructions as possible

- Try to match the size of the gap between the lines with the size of the objects in front of you, trying to think about the fact that your hand may seem smaller if it is taped together which may distort how the object looks.
- I was informed of the effects various factors can have on human perception of distance, such as tiredness, and other objects in the vicinity. I was than told that my left hand would be taped up in order to make it feel smaller. I was then told that I would put either my right or left hand through a curtain to pick up an object, and using the same hand I would estimate the size of the object on a computer screen by using the up and down arrow keys to manipulate two thin rectangles. These rectangles represented the vertical sides of the square.
- To reach under the curtain using the hand specified to retrieve a square shape. To then estimate the width of the shape and show the estimate by using the up and down arrows on the keyboard to change the line length. When doing so I had to take into account my right hand feeling smaller. I had to change the length of this line by pressing the arrows with the same hand I picked up the shape with. I then had to post the shape back through the curtain.
- To pick up the objects with either hand instructed. To decide on the size of the shape using my hand with my left hand taped up. Then using the arrows show the size of the object on the screen.
- Pick an object with either left or right hand (right hand was taped), estimate how big the object was on a computer screen. Put the object back into the box.
- You had your left hand taped up. Squares of different sizes were put behind a black curtain and you would use a different hand each time to pick up a square and use the lines to match up the width of the square, you had to use the same hand to press the keyboard and to pick up the square. With your left hand you had to include whether your hand felt small in the estimate of the width of the square. Then you had to draw around both hands, firstly with the left hand taped up and then without.
- I was asked to cellotape my right hand together, first my middle finger and my ring finger and then all four fingers together. I was asked to picked up various sized squares from under a black cloth and then demonstrate how large I through the shape was through an online slider. I was asked to take into account whether my cellotaped hand gave the impression that a square felt smaller or larger in my right hand compared to my left which had not be cellotaped together. I switched between my left and right hand when picking up the squared and then used this same hand to illustrate the size on screen.
- Using the arrows in on a keyboard you will move two lines to match the width of a square seen physically. With one hand taped up to give the illusion that the hand has been shrunk as perception of the size and object can be altered through various different factors like how tired one is, how hungry or energetic they are also. These can make these appear larger or smaller.
- Right hand taped up, presented with square stimuli behind black cloth, either instructed left or right, and the aim was the match the touch stimuli to two lines on a computer screen using up/down keys and state whether using taped up hand (right) made objects feel smaller
- The fingers of my left hand were taped together. Then I had to pick up squares of different sizes from behind a curtain with either my taped left hand or right hand and estimate the width of the square by moving two lines on a screen. The hand you picked up the square with was the hand you had to adjust the lines with.
- I had to pick up a square with either my taped or untaped hand and then estimate its width on the tablet screen using the same hand to change the width with arrow keys.
- Going to be picking up objects and estimating the width of them on a computer by using the arrow keys. The arrow keys make the lines on the screen move. One hand will be taped up to see if this makes your estimates smaller.
- Reach through the curtain to pick up the square stimuli with either your taped hand (right hand) or your non-taped hand (left hand) as instructed and place it on the table next to you. Use the same hand you used to pick up the square to press the up and down keys to change the distance between the two lines on the screen until you are satisfied they match the width of the square stimuli. Please keep in mind whether you feel like your right hand is smaller.
- To draw the width of the square object given to you through a black screen with either your right hand or left hand. The left hand was covered in tape therefore had to include whether your left hand felt smaller during the experiment.
- When the researcher says 'right hand' put your right hand through the curtain, pick up the object and estimate on the computer how big that object is by moving two lines on the computer screen with your right hand. The same for the left hand (which was taped together). I had to keep in mind whether the left hand felt any smaller.
- I had to reach for a square shape behind the curtain with either my left or right hand, put it beside me and then estimate the size of it by using some markers on a screen whilst taking into account how smaller or larger the shape felt when my right hand was taped.

1. What do you think the overall aim of the experiment was?

- To test perception and how changing something close to the object may change how the object is seen.
- To see if telling me that my hand would feel smaller would subliminally make me perceive it as feeling smaller
- To see how the size of your hand impacted on your estimate of the shape width
- To see whether my perception of the size of the card changed depending on what hand I used.
- To test whether having your hand cellotaped will make objects seem smaller than they actually are as your hand feels smaller
- To measure your ability to work out the size of an object when you believe that one hand is smaller than the other
- To see whether taping fingers on a hand makes us think that the objects that we pick up are bigger/smaller.
- To see whether when the hand is made smaller, whether the square stimuli also feel smaller because when your hand is free to feel the object you get a better idea of the size as you have both a visual idea and you can feel it.
- To discover whether the illusion of a smaller hand changed the way we perceived the size of a shape in comparison to our normal hand.
- To see how effective people are at judging and estimating size when a physical change has occurred and whether this affects judgement or not.
- I think the overall aim was to explore gender differences and hand sizes/spans/width in touch/perception of square stimuli
- To investigate whether having a constricted hand influences the size objects?
- To see how my perception of the size of objects changes when the task is more difficult to perform.
- To see how changing something about yourself impacts your perception of the width of objects.
- To investigate visual perception of size through comparison (i.e. you compare the size of your hand to the size of the square and this may change your perception of the size of the square)
- To see if the width of your hand changed your estimation of the width of an object
- To see whether our perception of objects is affected when our hand feels smaller
- To see whether people estimate objects smaller or larger when they had restricted access, i.e. grip.

1. What do you think the main manipulation of the experiment was?

- Whether or not your hand was taped together.
- Telling me my hand would feel smaller
- The size of the hand using the tape
- The celotape around my left hand.
- The tape on the fingers of one hand
- Not being able to see the object and that I could only pick an object up with one hand.
- The hand being taped up because you weren't able to feel the square stimuli as easily as with a free hand.
- Having one hand seem smaller than the other.
- The size of the hand.
- I think the main manipulation was cues from researcher in regards to if something feels smaller, how inclined participants may be to go above this when using right hand or vice versa
- Whether your hand was taped or not
- The person conductiong the experiment telling me to look if my right hand seems larger.
- Taping one hand together and telling you that this is supposed to make your estimates smaller
- The size of the square stimuli
- Taping the fingers together
- Whether the hand was taped together or not, whether the left hand felt smaller
- Taping the right hand.

5. What did you think was specific in your instructions? Did anything stand out to you?

- Remembering that it was important that the hand was either taped together or not, and how that affects how the size of the object feels.
- Telling me to imagine a house at the end of a street and how various things can affect how I perceive the distance
- Using the same hand on the keyboard.
- To remember to measure the size of the block with my hand
- Only picking the object up with one hand.
- You were told to include whether your hand felt small in the estimate of the width which stood out the most.
- That I had to use the same hand that had picked up the square to then demonstrate the size of the square on the computer.
- The detail in which is was described the examples used also.
- The instructions were clear, concise yet more complex than the actual computer task was made out, the tape obviously stood out as well as cues in perceived length , does it feel smaller if hand tape
- Which hand you had to pick up the object with
- To look at the size of my hand and whether to use the right or left hand.
- That taping your hand together would make your estimates smaller. This was reiterated inbetween the two tests
- Repeating 'please keep in mind whether your right hand feels like it’s smaller' and 'please use the same hand to type the keys'
- That you had to remember if the tape made your hands feel smaller
- The researcher repeated 'notice if your left hand feels any smaller'
- Straight forward, simple to understand.

7. What effect do you think the instructions had on your behaviour or responses? Please be as specific as you can, including the direction of any predicted effects.

- I thought more about how the size of the object feels compared to my hand and that I may think the object is bigger than it actually is because my hand was taped together.
- Telling me that I could only us the arrow keys with the specified hand made me actively monitor which hand I used.
- I felt that I should be giving larger shape size estimates for when I picked up shapes with my right hand which was taped
- I believe I made the objects width larger as I thought my hand felt smaller
- Not a clue!
- Being told one hand was smaller than the other
- The instructions made me feel like I should think that the objects in my right hand will feel bigger than what they actually are.
- I thought that being told to include about if my hand felt small in the estimate made me think about how big I made the width, in some cases I felt like the width wasn't as big because you couldn't feel all of the square.
- That any shape held in my cellotaped hand would seem smaller as I felt this is what was expected.
- At first, I thought the taped hand made the objects seem smaller but when I thought about it, the change made the objects appear bigger than they are. If the researcher had not said to include if the fingers taped made the object feel smaller I may not have realised this.
- I think the instructions (cues) were made to see how easily influenced/perceptible people are to changing answers, taking longer when told things feel smaller when hand taped up etc, so when picking up things with taped hands, participants may have felt they were feeling it smaller than it was and therefore overestimated with said hand
- I looked at my hands more, however I think the object size seemed the same so I tried to answer as accurately as I could.
- Being told that having my hand taped together would make my estimates smaller made me think a lot more about what I was estimating with my taped together hand
- I assumed I had judged my right hand squares to be bigger than they actually were, so I may have altered my initial opinion and said they were smaller. Vice versa with the left hand
- I tried not to let the tape alter my predictions and did this by placing the objects on the table to estimate the width rather than feeling them with my hands
- I had in mind that my left hand felt smaller but I’m not sure if this actually made me estimate the squares smaller when I used my left hand.
- Yes I was conscious of my answers more due to being told to take into consideration whether the shapes felt smaller or larger with the taped hand.

9. What effect do you think that taping your hand had on your behaviour or responses? Please be as specific as you can, including the direction of any predicted effects.

- Made the objects look bigger compared to my hand when it was taped up rather than when it was not taped up.
- I was afraid of moving my hand too much in case I ripped the tape off. It also made me be more careful while grabbing the shapes from behind the curtain.
- Yes I felt that I should be giving larger size estimates with my taped hand
- Not a clue!
- Made the object seem smaller
- The right hand was different to the left, so I felt like the results are going to be different e.g. the object in the right hand will feel bigger.
- Yes it made me put the width of the squares as smaller in some cases because you couldn't feel all of the object.
- I think it gave the impression that the square held in this hand felt differently sized to when held in my non taped hand.
- It made the appearance of objects seem larger which may of affected my estimations.
- It made me think and question my responses more in that the researcher said things feel smaller in taped hand in that picking up a bigger thing mainly I wanted to ensure I made it smaller than I think as I assumed I’d naturally over estimate it, as took the researchers word and am obviously very impressionable
- Maybe a smaller estimation as you couldn't really hold the object
- I do not think it did.
- It made me think harder about my estimates with that hand
- It made my hand feel like it was smaller
- It made me more cautious but I tried not to let it influence the width
- It made me more aware that my hand might have felt smaller becuase it was taped together but I’m not sure if this affected how I estimated the objects, I tried to be as accurate as possible
- I think it made the shapes feel larger.

10. Having answered these questions, what do you think the overall aim of the experiment was?

- To look at how instructions may change perceptions.
- To see if telling participants that the tape will make their hand feel smaller will affect how they perceive objects
- To see how taping the hand and prompting the participant to take into account how much smaller it felt would influence the person's responses of shape width in terms of them thinking they should give larger estimates for this even when it did not actually feel this way
- Not sure. Possibly to do with perception and restricted motion?
- To see if being told one hand was smalller influenced the estimation of the size of the shape
- Checking whether instructions can influence how big the object will feel in the hand.
- That having your hand taped up makes you estimate the width of the same size objects different as your hand feels smaller than the other, making you estimate that the object is bigger than what it was.
- To find out whether our perception of something up close is able to be manipulated,
- To see the effect of a change in hand size affected the estimation of the size and to see if by leading the participant into thinking the change made the object seem smaller affected their estimations of the object also.
- To investigate whether a constricted hand influences the estimation of the object
- To see if the instructions of the experiment would make me behave differently giving my answers.
- To see if telling someone that something will affect them makes them change the way they act and do what they were told would happen
- To investigate social desirability and whether leading instructions have an effect on the results of the study
- To see if telling someone their hand may feel smaller would alter their perception on the width of an object
- To see whether reminding someone that their hand feels smaller actually affects the way they perceptive the size of their hand
- To see whether the instructions given affect how individuals estimate shape size when one hand is restricted, i.e. taped.

**Experiment 2: Action capacity – direction specified group**

1. Please try to recall the instructions of this experiment in as much detail as you can, and write what you remember below. It is important that your description provides as much detail of the instructions as possible

- To estimate the size of the square which I had to pick up using either my taped right hand or untaped left hand. I had to remember to take into account outside influences which would affect my judgement of the size of the object e.g right hand taped would make me think the square was larger.
- I had to pick up the square from under the sheet with either my left or right hand and place it down at the side of me. I then had to estimate the size of the square, as if I was measuring one of it's edges with a tape measure, by using the software programme. This had to be done by using the same hand I picked the square up with, using the keyboard arrow keys.
- To pick up sqaures and estimate the size of them, bearing in mind how big they felt to pick up in the taped hand and untaped hand.
- To when asked reach through the curtain in front of me to collect an object either with my taped right hand, or with my free left hand, which hand I needed to use was specified by the researcher.Then I would use the up and/or down arrows on the keyboard to estimate the size of the object that I had picked up with either my left or right hand.
- To pick up square objects with either my left hand were my fingers will be taped up or my right hand, then use the up and down arrows on the keyboard, make the lines that appear on the laptop screen the same width as the square object taking into account whether the object feels bigger or smaller depending on the hand I picked it up with
- Firstly I had my fingers on my right hand taped up, my ring finger and middle finger were taped first and then all 4 fingers were taped together. I was also told that we generally estimate the size of objects larger than they actually are because we are clumsier with our fingers tied together. I was then asked to pick up square stimuli and using arrows on a computer, I estimated the distance of the squares. I was asked to alternate which hand I picked up the squares with at random and completed 20 trials in total
- To pick up the square from behind the curtain with either my left or right hand, which I was told my right hand was taped up. I then had to try and change the distance between the lines to match the size of the square that I had picked up and to consider how easy it was to pick up.
- Pick up the objects, with either your right hand or your taped left hand. Match the lines in the screen to the size you think the square is. Always think about how difficult it was to pick up the object and whether it was more difficult with your taped hand.
- The instructions of the experiment were to estimate the size of the squares that I had to collect through a black curtain. I estimated their size by changing the distance between two lines on the screen using the up and down arrows on the keyboard. I had to pick up the squares using either my left hand or my right hand that was taped and then use the arrows to change the size with the same hand.
- I was told the instructions of the experiment and then my ring and middle finger on my left hand were taped together and then all four of my fingers were taped. I then had to pick up square shaped randomly using my left and right hand. Once I had picked up a shape with one hand I was told to place it on the table and use the arrows on the keyboard with the same hand I had picked the shape up with, in order to adjust the two parallel lines on the screen to match the size of the shape next to me. I had to do this 10 times in each of the 2 intervals.
- Use taped hand to see if objects appeared larger than in non taped hand
- To pick up the given squares in the instructed hand and guess the size of the object by moving the lines on a computer programme to match how big you think the square is and to press enter when you percieve how far the lines are apart as the same as the width of the square
- Going to pick objects up with either your left hand (untaped) or your right hand (taped), you then using the lines need to say how big the object was (felt).
- My right hand was taped up (four fingers), and I had to reach behind a black curtain with each my right taped up hand or my left hand and retrieve an object. Then I had to try and guess the size of each one via a computer programme and could adjust this with the up and down arrows. There were 2 groups of 10 trials and my left or right hand was randomly chosen by the experimenter to select each item.
- One hand taped, one not. Pick up an object with instructed hand and then use the computer programme to move two lines to match the width of the object. Take everything about picking up the object into consideration, especially how difficult it was to pick up.
- Picku p the different objects with the different hands randomly, use the same hand to change the size of the lines on the screen.
- At the start of the experiment the fingers on my right hand were taped together and my left hand was left un-taped. I was asked to reach through the curtain with either my right or left hand and to pick up a white square of various sizes one at a time. Once I had picked up the square and placed it next to me I then had to estimate its size using a computer programme which allowed you to either increase or decrease two lines to try and match the size of the square. On each trial there were 10 attempts and in total there were two trials. After the trials were finished I had to draw around both of my hands and then again on my right hand once it was un-taped. That was the end of the experiment.
- Put your either your right hand or left hand (the hand that’s taped together) through the curtain and pick up a square. Keep in mind whether objects feel bigger in the hand that’s taped together. Then use the arrow keys to move the two lines on the screen to match the width of what you think the square is that you just picked up. Then press enter and pass the square back through the curtain.

1. What do you think the overall aim of the experiment was?

- Maybe to see that if I am told my judgement would change when I pick it up with my right hand regardless of if I think its the normal size.
- To investigate whether I perceived the size of the squares differently when using the right hand compared to the left hand which was taped up.
- To see whether objects appear bigger in different situations, so whether the object seemed smaller when I could make my hand bigger than it did when my hand was taped together.
- To see if having a taped hand meant that I would over estimate the size of the object compared to when I would estimate it with my left hand.
- To see whether the inability to pick up an object with normal use of fingers makes the object perceive bigger or smaller
- To see if i had higher estimations of the size of the squares with my fingers taped together
- To see whether or not the dominance of the hand influences how we perceive the size of objects or not?
- To see if we perceive objects as being bigger if we find them more difficult to pick up.
- To see if having my hand taped affected my perception of the size of the squares
- To find out whether picking u a shape with the hand I don't write with taped together made it harder to work out the size of the shape than with my normal right hand.
- To see if objects appeared larger when using taped hand
- To see if your hand being taped together made you percieve the objects as bigger
- Whether having your mobility restricted in your dominant hand affects your ability to judge the size of the object.
- To see whether objects picked up with a taped hand are perceived larger than those picked up with an untaped hand.
- To see if perception of how difficult it is to pick up an item effects how wide we perceive the item to be.
- To see if telling me that the taped hand makes a difference to perception affects my judgement
- To see if the perception of the size of objects changes depending on the surrounding environment
- To see whether we perceive objects as larger when they are more difficult to pick up.

1. What do you think the main manipulation of the experiment was?

- The size of the squares
- The left hand being taped up.
- Changing whether or not I could use my hands properly
- Taping the right hand.
- Whether the fingers of the one hand that were taped made a difference to how I perceive the size of the square object
- Having our fingers taped together and being told that we usually have larger estimations when we are clumsier
- The hand used to pick up the object
- The size of the squares I had to pick up.
- My right hand that was taped
- I feel the main manipulation of the experiment was the taping together of my fingers on my left hand.
- The sizes of the blocks
- Whether the taped hand or untaped hand picked up the square
- Whether it was your right hand or left hand used.
- Taping the right hand up
- The size of the object.
- Whether the hand was taped or not
- The size of the object
- Whether the hand used to pick up the objects was taped together or not.

5. What did you think was specific in your instructions? Did anything stand out to you?

- Told to remember to take other influences into account and asked to repeat the instructions back to the experimenter
- Things had to be done with the correct hand and the size adjusted on the laptop with that same hand.
- That estimation of size of objects may vary if one hand is taped up whilst the other is free.
- That I was going to be picking up square objects with a taped hand to see the difference between my untaped hand
- The fact that we were told we have larger estimations when our hands are taped together
- To pay attention to how easy it was to pick up
- The detail given of the background to the aim of the study.
- It was clear that I had to use the same hand to pick up and manipulate the size of the squares
- The fact that my fingers being taped together could affect the way I perceived the object stood out to me.
- Using only the instructed hand at one time
- That it's thought that the taped hand would estimate objects as larger
- What hand you were going to use and that same hand used pick up the object was one you had to use to move the lines
- Just how clear and specific the instructions were. Also that two of my fingers were taped first and then the four fingers together.
- Consider whether or not the object was difficult to pick up when deciding how wide it is.
- That I was told the taped hand would make the object seem larger to me
- When estimating the size of the square, to be fast but as accurate as possible
- That I had to think about whether my hand being taped together made a difference to how I perceived the size of the object.

7. What effect do you think the instructions had on your behaviour or responses? Please be as specific as you can, including the direction of any predicted effects.

- I was told that if I have my right hand taped it would make me think the square is bigger. I think this made me make it bigger even if I thought it was smaller
- I think they made me focus on the task given. I don't think they affected me in any other way.
- Probably made me estimate bigger sizes for the objects that felt big to pick up with my taped hand.
- As I was told to keep in mind that there may be an effect on estimation I spent longer making sure that the objects were definitely the size I perceived them to be and not just estimate them based on what the instructions had said.
- I was more aware of thinking that the object seemed to be bigger when using my left taped hand
- I tried to see the squares as larger than they actually where, therefore the distance I made the lines on the computer may have been further apart than they should have been
- It made me more aware of which hand I was using and how easy it was to pick up, it also made me think more about the actual size of the object when trying to match it to the screen.
- It made me aware that I might perceive the objects I picked up with my left hand to be bigger so I tried to be very careful when matching the lines on the screen to the size I thought the square was.
- The instructions made me more aware that the experimenter was looking to find a size difference (larger) when using my taped right hand. This could have possibly cause demand characteristics in other participants.
- I feel that I may have thought more about the shape when I was picking it up with my left hand as I was curious to see if I did perceive it differently.
- Being aware that when using my taped hand I would say that the objects were larger
- I may have estimated the objects picked up with my left hand as bigger
- Made me more or which hand I was using, and that the size of the object should feel bigger when using your taped hand
- Because I was told that it is suggested that the taped hand would perceive objects as larger, I think I tried not to do this.
- I think I thought more about how hard it was to pick up each item and this may have made me overestimate the width of the objects I picked up in my taped hand.
- It made me made sure that my judgement wasn’t affected by what the researcher said however, if anything it made me underestimate my left hand guesses.
- To increase the size of the object when using the hand that had be taped up
- I think they made me over-think my responses and possibly exaggerate my answers when using my taped hand - i.e. perhaps I increased the width between to the two lines more when using my taped hand than I did when using my non-taped hand

9. What effect do you think that taping your hand had on your behaviour or responses? Please be as specific as you can, including the direction of any predicted effects.

- I think it did make me think the squares were a bit bigger but I still exaggerated the size of it because I was told to
- Unconsciously, think it made me feel like the squares were larger when using that hand.
- It made objects feel bigger as my hand span was smaller
- It meant I tended to over reach for the objects rather than just pick them up like normal. It also made me question how large the objects were.
- I thought about perceiving the square objects as bigger and expected them to be difficult to pick up
- I found it easier to pick up the squares with my hand taped although it did provide me with a distraction as I was more focused on how clumsy my hand felt
- It made me more aware of what the hand was doing as I had to pay more attention to it rather than it just happening from memory
- I was being a lot more careful with my taped hand especially when picking up the objects as I felt I had lost a lot of control over it. I also tried to accurately match the lines to the square being more aware of how difficult I found it
- It made me more clumsy when picking the square up making things difficult but I don't feel that it made me perceive them as larger than they were
- It made me think more about what I was doing although I am unsure if it actually affected the way that I perceived the object.
- I was aware that there could be a difference
- Yes because I struggled to pick up the bigger squares with my left hand so they seemed larger so I may of predicted them as bigger
- That when I picked an object up with that hand it should feel bigger and more difficult
- I tried not to make objects seem bigger than they were
- It made me presume the item was larger than it was (I think)
- I do not think it had any effect on my behaviour
- I knew that with a taped hand the objects would feel bigger than with the un-taped hand, therefore I probably guess greater estimates for the size of the square on those trials
- I think it made me perceive objects as bigger, but I’m not sure to what extent.

10. Having answered these questions, what do you think the overall aim of the experiment was?

- To see participants would change the shapes to be bigger when using the right hand to conform to the experimenter
- To see whether I estimated the size of the squares differently when using my right hand compared to my left which was taped up.
- To see how we perceive the size of things
- To see if using instructions containing "hints" or the desired aims of the experiment would impact the outcomes of the experiment.
- Whether being told that an object will seem bigger when your hand is taped will make the object actually seem bigger
- To try and see if the participant would show demand characteristics
- To see the effect of hand dominance on how we perceive things
- Whether you will find it harder to pick up objects and accurate estimate the size of them if you have been told it will be more difficult if your hand is taped.
- To see if certain obstructions (like having my hand taped) skews human perceptions
- I think that the overall aim of the experiment was to see if telling me that the experiment was looking to see if taping my hand together could effect the way that I perceive objects, would make me change the way that I do perceive the object during the experiment.
- To see how the wording of instructions could influence behaviour
- To see whether having a taped hand effected the percieved estimated size of the square
- Making you believe that having your hand taped will affect your ability to judge its size, this is how you'll perform in the study
- Whether or not I was influenced by being told at the start whether taping a hand perceives objects as larger
- To see if our ability to pick up items affects our perception of their size.
- To see if telling me taping my hand would make a difference to my judgement would actually influence my answers
- To see whether our perception of objects changes depending on the surroundings
- To test whether object are perceived as larger when they are more difficult to pick up.

**Experiment 3: Report graspability group**

1. Please try to recall the instructions of this experiment in as much detail as you can, and write what you remember below. It is important that your description provides as much detail of the instructions as possible

- One of my hands would be taped together, and the other one would be free. The researcher would then instruct whether I would reach for thee object with my right or left hand, and then using the up or down arrows I would have to estimate the width of the object with the same hand I used to grab the object, after instructing on a scale of 1-10 how hard I found it to grasp the object.
- Grasp the object / Bring it in front of self / Express whether the grasping was easy or not, taking into account the hand used (whether taped or not) and whether the physical restraint of the hand plays a role in the grasping judgment / (try to) match the physical (perceived) width with the computer matching task
- We are going to tape up your right fingers and I want you to put your hand through the cloth and try and pick up the object with the hand I tell you to. Then rate how hard it was to pick up. Then using the arrows and the laptop program to estimate the size of the object and pass the item back through the cloth then we will do some other tasks after 2 trials of 19 rounds
- First of all I had to tape up my left hand with selotape. Then I had to pick up objects with either my left or right hand and say how difficult it was to pick up these objects then I had to guess the length of the sides of the objects and use the laptop to drag out the two lines to get the right length. I did two lots of 20 trails of this. Then I had to draw around my taped up hand, then around both hands with no tape on them
- Going to have the fingers on one hand taped together and behind the curtain there will be an object placed that I need to grasp. I need to grasp the object with my thumb on one end and the rest of my fingers on the other end of the object. Then, on the computer, I need to adjust the lines with the arrow keys so that they match the size of the object. After this is done, I need to pass the object back through the curtain with the hand I picked it up with .
- Estimate the size of the squares and state the difficulty of picking up each square with one hand free and the other taped
- With one hand tapped, reach behind curtain and pick up object, rate on scale of 1-10 how difficult object was to pick up (taking into account more than just visual cues)
- With one hand taped together and the other not, reach through a curtain to pick up an object. The place it on the table and on the computer estimate the size.
- Estimate the difficulty to pick up and the size of objects as you attempt to pick them up with either your non dominant hand, or your dominant hand with fingers taped together.
- One hand will be taped and you must grab objects from behind a curtain. Say how hard it was to pick object up with R or L hand. Change width of lines apart using arrow keys to match size of object picked up.
- My right hand was taped together and then I was asked to pick up squares through a curtain with either my left or right hand and then record how hard I felt it was from 1-10, 1 being the easiest, and then estimate the distance between the two lines on the screen in terms of the size of the square.
- From a range of 1-10 I had to rate how hard picking up a square was with either a taped up hand (left hand) or right hand.
- I had to describe the level of difficulty in picking up objects behind the black screen. I had to rate my level of difficulty between 1-10. 10 being very difficult and 1 being the least. The researcher emphasized that it would be harder for me to pick up the objects with the hand that I had celotaped together (right hand)
- My middle and ring fingers were celotaped together and then my index and small finger were also taped with the ring finger and middle finger. I was asked to pick up items from behind a curtain with my celotaped hand and my free hand and had to say how easy it was.
- The instructor would say left hand or right hand, then I need to use that hand to take a square object. Then I have to answer how hard does it take and estimate the length of it on the computer.
- The aim of the experiment was to see how distance affected the size and perspective of different objects. Objects were placed behind a screen and using one hand normally and the other taped I had to sit behind a screen and feel objects, estimating the distance between their length on between 2 parallel lines on the computer
- To pick up squares with either my right or left hand (my left hand being taped up) and then guess the width of the object once picking it up. Make the lines on the screen the true width of the size of the square picked up using the up/down arrow, also using the same hand that the object was picked up with.
- I had to put my hand under a black sheet, the hand that the experimenter told me to use, and I had to pick up an object and rate how difficult I found it to pick up (1 being easy, 10 being hard). I then had to use the same hand to move two lines apart to match the size that I thought the object was

1. What do you think the overall aim of the experiment was?

- To see whether taping one of your hands would make it harder to guess the estimated size of the object.
- Well makes me think of whether the explicit physical restraint of the hands plays a role in the explicit judgment of the "grasping easiness"; another thing to consider is whether the explicit judgment has an influence in the matching task (i.e., if I judge the grasping of a square "easy", do I also judge its width as shorter than it really is? And so on)
- To measure how using your non dominant hand/side changes perception
- To see if peoples perceptions of size of objects changed when it was more difficult to pick up the object due to the taped hand
- To determine whether or not having fingers taped together affected the level of difficulty it was to pick up the object and also whether or not the size of the object made affected the level of difficulty it was to pick up the object.
- To see if the difficulty of picking up the objects affected the size estimated
- To see if having one hand tapped affects how difficult you perceive picking an object up
- To see whether restriction of hand and finger movement affected ability to pick up an object and accurately tell its size.
- To see whether your estimation of the size of objects increases with the difficulty of the task involved.
- To see how restrictions of hand span affect estimation of size of object.
- To discover the implications of my dominant hand being less easy to use and how that affected my perception of the space between the lines.
- To see which hand we find easier to pick up objects that we cannot see.
- To see if the lines were more apart when I had to pick up the object with the celotape (Right hand) and see if the lines were more closer together with my left hand.
- To see how things are perceived
- To see the difference of feeling the object when one hand is tight and one hand is relaxed. Also investigate would the obstacle that block my hands would make me feel harder and affect the estimation.
- To see if there was any difference between psychological factors on distance with objects
- To see whether perception of the width of the object was influenced by its size/ difficulty to pick up
- To see different peoples perception of object size and also to see whether having your dominant hand taped up affected your ability to grab the objects

1. What do you think the main manipulation of the experiment was?

- Not seeing the object before you grasped it
- Object width
- The use of non dominant hand
- Either having a free or taped up hand
- The size of the object.
- Taping the fingers together makes you believe it is harder to pick up the squares
- Having a taped hand
- Whether the hand/fingers were taped up.
- Whether the fingers of the dominant hand were taped together or not
- Whether the hand was taped or not.
- My hand being taped.
- Not being able to see where the objects were
- The researcher emphasizing that I would be more restricted with my right hand
- Not too sure
- The taping and the setting of table
- The restriction of the use of one hand, made it somewhat harder to grasp some objects
- Saying that it is based on how difficult/ easy it is to pick up
- The size of the objects

5. What did you think was specific in your instructions? Did anything stand out to you?

- Whether I needed to use my left or right hand
- Take into account the physical restraint in your judgment of how easy it was to grasp it
- How to complete the study, clear instructions of what to do
- The researcher made read out intructions that she had already written so they were specific and every participant would hear and understand the same instructions
- The fact that the hand I used to pick up the object also had to be the hand I used to press the keys on the keyboard and pass the object back through the currtain.
- The instruction to give the estimation of the object was given multiple times, with a reminder that the taped hand should find it more difficult to pick up the square
- Don’t just take into account the visuals from the object
- How to pick up the boxes.
- Yes the instructions were specific - the ones that stood out were to complete everything with the same hand, and how to pick up the blocks
- The number scale.
- The way in which I was to pick up the objects and which hand I should use
- The instructions hold me that one of my hands would be taped up and what I had to do to complete the tasks
- Nothing was really specific
- That I could only pick up objects by holding them from the sides
- How to perform the required activity. It was all pretty clear
- Saying you must use the same hand to respond with and also that you must answer based on the difficulty of picking up the object whether your hand was taped/non-taped
- I was instructed where to put my hands and what kind of object to grab and also how to use the computer programme and that I had to use the same hand I picked up the object with to move the lines apart on the screen

7. What effect do you think the instructions had on your behaviour or responses? Please be as specific as you can, including the direction of any predicted effects.

- No effect
- I think I tried to make the matching task (i.e., match the two vertical black bars to the perceived width of the physical square) "as if" the more difficult I perceived it the grasping to be, the more wider the bars should have been. In other words, the instructions could have created a "bias", an "expansion effect" were when the object was grasped with the physically restrained hand, there was a systematic enlargement of the bars regardless of the difficulty.
- I rated the difficulty of picking up the squares as harder for the taped hand than the free hand every time
- Without the instructions my ratings would have been more based on the size of the object visually as oppose the weight or which hand was being used
- I would have picked up the boxes differently otherwise.
- Made me give specific numbers and use the correct hand when altering the lines.
- They directed me in what to do and allowed me to attempt to predict which hand would be asked to pick up the square next, however I was not always correct
- Due to one hand being taped up you instantly think that is your weaker hand so would rate higher out of 10 when using that hand
- Perhaps because of the inital description about how judgement was different depending on feelings. I tried to be as accurate with both hands
- To concentrate on picking the object up from the sides
- I think the main behaviour I had was to be honest, and my results were an honest representation of what I could feel and see
- I don’t feel as if they influenced my responses, I responded truthfully
- I kept having to think about which hand I was being told to use and also had to make sure I used the same hand to move the lines apart. This may have affected me because it might of made the thinking processes more confusing

9. What effect do you think that taping your hand had on your behaviour or responses? Please be as specific as you can, including the direction of any predicted effects.

- Found it much harder
- I think it did according to what I wrote before (taped hand = "expansion effect") but in reality I think the effect of the tape was less than the effect of the instructions itself. This is mainly due to the fact that the square grasping task wasn't excessively affected by that.
- It made me more aware of my concentration to pick something up
- I think it made it more difficult for me so maybe I then overestimated the size of the object
- I think that because my hand was taped together I expected that picking up the object would be harder and so gave a higher score for these conditions.
- The taped hand made the object feel harder to pick up and often larger than it really was
- The taped hand made it more difficult to pick up the objects therefore I rated them higher
- Some were harder to pick up and may seem bigger than they were because of this.
- I think picking up the objects would have been easier with my right hand than my left hand if my fingers hadnt been taped together, so the estimation of object size would have been more accurate
- Affected the distance the lines were apart.
- It made me think that it will automatically be harder for me to pick up the objects
- Increased the number that I gave
- My taped hand always crash the upside down table in front of me
- Taping the fingers made it somewhat harder to grasp some objects but not drastically
- I don’t think that it had an affect, I thought that it may had but actually it didnt affect me picking the objects up any different than the non-taped hand
- It made me think that it would have been more difficult to pick up the objects thereforeI rated it as being more difficult

10. Having answered these questions, what do you think the overall aim of the experiment was?

- Behaviour responses in grasping the object with a taped hand
- Hmmm to see whether the instructions have a more influential effect on the subject compared to the main manipulation (squares, or taped hand)?
- To see if using non dominant side influences perception of vision/size of objects
- To find out if making it harder to pick up an object would make a person over estimate its size
- To see if the difficulty of picking up the object influenced how well I estimated the size of the object on the computer screen.
- To see if the difficulty of picking up the objects affected the size estimation (more difficult objects = larger)
- To see if other factors influence our perception of objects as oppose to just visual cues
- To establish whether restricting hand/finger movement affected how easy boxes were to pick up and estimation of how big they were.
- To show that the difficulty of a task influences visual perception of objects
- To see if a restriction of hand span affected estimation of shape width.
- To see if taping my hand affected how I thought about the size of the shape and to see if the specific instructions would make me predict which hand would be used next
- Predicting object size
- Still unsure?
- Not too sure
- The aim is to investigate the difference of feeling between relax hand and taped hand
- To see whether a persons view of an object in a distance is different based on different factors influencing it. For example, in this case, taping the fingers on one hand and the other hand being free.
- To see whether the hand being taped had a percieved effect on the objects width, the harder it was to pick up the larger you’d change the width to on the computer.

**Table 7**

Responses to Q4 of the online post-experimental questionnaire or each group in Experiments 1, 2 and 3, given as n (%).

|  | Action Capacity group  (Expt 1,  n=18) | Objective  Size group  (Expt 1,  n=18) | Body  Size group  (Expt 1, n=18) | Action Capacity - Direction Specified group  (Expt 2,  n=18) | Report Graspability group  (Expt 3,  n=18) | Total  (n=90) |
| --- | --- | --- | --- | --- | --- | --- |
| Agreed that the instructions given were very specific | 17  (94%) | 17  (94%) | 18  (100%) | 18  (100%) | 16  (89%) | 86  (96%) |
